# Engineering Multifunctional Peptide-Decorated Nanofibers for Targeted Delivery of Temozolomide across the Blood–Brain Barrier

**DOI:** 10.1021/acs.molpharmaceut.4c01125

**Published:** 2025-03-17

**Authors:** Rosa Bellavita, Teresa Barra, Simone Braccia, Marina Prisco, Salvatore Valiante, Assunta Lombardi, Linda Leone, Jessica Pisano, Rodolfo Esposito, Flavia Nastri, Gerardino D’Errico, Annarita Falanga, Stefania Galdiero

**Affiliations:** † Department of Pharmacy, School of Medicine, University of Naples Federico II, Napoli 80131, Italy; ‡ Department of Biology, University of Napoli Federico II, Via Cintia, Naples 80126, Italy; § Department of Chemical Sciences, University of Napoli Federico II and 4CSGI (Unit of Naples), Via Cintia, Naples 80126, Italy; ∥ CSGI (Unit of Naples), Via Cintia, Naples 80126, Italy; ⊥ Department of Agricultural Science, University of Naples Federico II, Via Università 100, Portici, Portici 80055, Italy

**Keywords:** self-assembling peptides, glioblastoma, nanofiber, blood−brain barrier

## Abstract

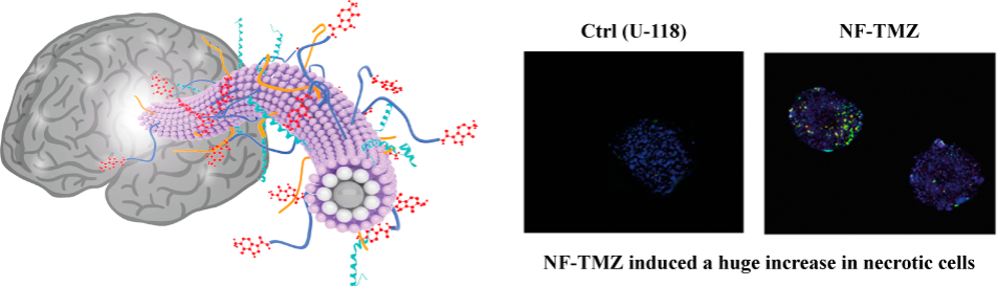

A nanoplatform based on self-assembling peptides was developed with the ability to effectively transport and deliver a wide range of moieties across the blood–brain barrier (BBB) for the treatment of glioblastoma. Its surface was functionalized to have a targeted release of TMZ thanks to the targeting peptide that binds to EGFRvIII, which is overexpressed on tumor cells, and gH625, which acts as an enhancer of penetration. Furthermore, the on-demand release of TMZ was achieved through matrix metalloproteinase-9 (MMP-9) cleavage. Nanofibers were characterized for their stability, critical aggregation concentration, and morphology. Next, the effect on both 2D and 3D glioblastoma/astrocytoma (U-87) and glioma (U-118) cell lines was evaluated. The Annexin V/Propidium iodide showed an increase in necrotic and apoptotic cells, and the morphological analysis allowed to discover that both U-118 and U-87 spheroids are smaller in surface, perimeter, and Feret’s diameter when treated with NF-TMZ. The developed nanofiber was demonstrated to permeate the BBB *in vitro* in a 3D spheroidal biodynamic BBB model. Finally, there were no cytotoxic effects of nanofibers without the drug on spheroids, while a significant decrease in viability was observed when NF-TMZ was used. Overall, these results open new opportunities for the evaluation of the efficacy and safety of this nanoplatform in *in vivo* studies.

## 1 Introduction

Traditional cancer therapies include surgery, radiation, and chemotherapy, although these treatments involve highly toxic compounds with severe adverse effects and the risk of incomplete cancer eradication.^1,2^ Recent research aims to develop next-generation anticancer nanomedicines,^3,4^ exploiting multifunctional nanoplatforms that can adapt in response to physiological, chemical, and physical stimuli.^5,6^ This strategy is important, especially for the most aggressive cancers like glioblastoma (GBM) where current therapies offer limited patient outcomes.^7,8^

GBM is the most aggressive type of brain cancer, with a 5-year survival rate of less than 5% and a median survival period of 12–15 months.^9,10^ The standard treatment, consisting of surgery followed by chemotherapy with temozolomide (TMZ) and radiation therapy, marginally improves survival. TMZ was approved by the FDA in 2005 and has been shown to extend patient life from 9 to 14 months when administered in conjunction with radiation therapy.^11^ Unfortunately, TMZ’s efficacy is limited by several challenges: i) poor water solubility and a short plasma half-life of only 2 h; ii) inadequate tumor accumulation due to the blood–brain barrier (BBB) and efflux pumps; iii) limited extravasation from blood to brain (∼20%); iv) low selectivity failing to differentiate between healthy and cancer cells.^12−14^ In GBM treatment, due to difficulties in total surgical resection and resistance to TMZ,^15^ a 95% chance of tumor recurrence within 7 months postdiagnosis is nearly certain. The use of high TMZ doses could potentially overcome resistance but causes severe side effects like myelosuppression and cardiomyopathy.^16^ These issues, combined with the tumor’s heterogeneity and barriers to drug delivery, result in poor survival outcomes. To reduce systemic side effects, address TMZ resistance, and improve patient survival, innovative, targeted TMZ delivery methods for GBM are urgently needed.^17^

Clearly, drug delivery ensures that the drug can be easily administered and delivered to the target tissue while minimizing side effects and optimizing therapeutic efficacy.^18,19^ Despite the efforts, delivery technologies encounter many obstacles:^20^ i) a reliable and strong drug loading that prevents early payload release to ensure therapeutic benefits with a low risk of side effects; ii) an in-depth understanding of the nature of the interactions between the drug and the delivery system; iii) the effectiveness of the controlled release allowing the drug to be released at the right time, with the right dosage, and in the right place; iv) control over the substance throughout its entire life cycle, from production to degradation and fate within the body; v) use of labeling tools that are simple to apply to track the drug delivery tool without changing its physicochemical characteristics. Overcoming these challenges requires multifunctional materials, guaranteeing precise control and on-demand drug release.

Therefore, we aimed to develop biodegradable materials for targeted and controlled delivery of TMZ for GBM exploring methods such as intermolecular interaction forces, where thanks to reversible self-assembly the delivery tool can be degraded into small molecules that can be more easily excreted or cleared by the organism. Specifically, we developed a delivery system based on the self-assembly of amphiphilic peptides to attain nanofibers decorated on their surface with several moieties, including targeting, penetration-enhancing, and therapeutic entities. Through a variety of intermolecular noncovalent interactions, such as hydrophobic contacts, hydrogen bonding, π–π stacking, metal–ligand complexation, and van der Waals forces, peptides spontaneously arrange and form structurally distinct and stable nanostructures through a relatively simple process known as self-assembly.^21^ These self-assembled nanosystems exhibit entirely different therapeutic capabilities from monomeric peptides, and they typically overcome the stability issues that occur with monomeric peptides to significantly increase their biomedical utility. Furthermore, the shape of the nanoplatforms obtained from the peptide assembly is a key determinant of *in vivo* fate.^22^ For instance, nonspherical nanoparticles show more effective internalization and present a higher surface area for multifunctionalization. Moreover, self-assembling sequences can be engineered to have the desired functions, which makes it simple to adjust their activity by altering only the quantity and/or type of the various moieties on the surface without affecting the self-assembled nanostructure. To enable self-assembly into highly ordered nanostructures, we designed a structurally defined nanoassembly made of two structural amphiphilic peptides (PAs), characterized by the presence of an amino acid sequence of aliphatic residues containing a lipidic tail (C19), and charged amino acid residues implicated in the peptide assembly. In addition, the developed fiber was functionalized on its surface with a cell-penetrating peptide, gH625, which we previously demonstrated to be able to enhance penetration across cell membranes and the BBB.^23−27^ The surface of the nanoassembly is further decorated with the targeting sequence falGea, which is able to recognize the overexpressed epidermal growth factor receptor (EGFRvIII).^28^ TMZ is covalently bound to the fiber surface through an on-demand strategy, which allows for drug release at the target site through a selective cut by matrix metalloproteinase 9 (MMP-9).^29^

A comprehensive experimental strategy combining fluorescence microscopy, electron paramagnetic resonance spectroscopy, and circular dichroism was used to characterize PA self-aggregation and the structural features of the resulting nanofibers. To evaluate the effect of NF-TMZ (nanofibers conjugated with the drug), we performed different *in vitro* experiments on GBM/Glioma cell lines (U-87 MG and U-118 MG) (ATCC) in both 2D and 3D cultures. By 2D studies and microscopy, we evaluated the most appropriate percentages of targeting (falGea) and cell penetration enhancer (gH625) that needed to be present on the NF surface to achieve cell uptake. Then, we evaluated the effects of NF-TMZ on 2D/3D U-87 and U-118 cells at different concentrations to determine the impact on cell viability over a period of up to 72 h. 3D U-87 and U-118 cells were also used to perform an Annexin/PI assay to discriminate, following treatment, the populations of necrotic and apoptotic cells. Furthermore, as the brain endothelium is a marker of EGFR expression, the effects of NF-TMZ over a period of up to 72 h were also evaluated on 2D HBMEC and bEnd.3 cells.

To better analyze the ability of NF and NF-TMZ to cross the BBB, we set up a dynamic 3D *in vitro* BBB model that reproduces physiological conditions. This model was also used to evaluate the viability and cytotoxic effects on 3D spheroids.

The developed platform is highly adaptable, with the ability to effectively transport and deliver a wide range of moieties across the BBB. The insights gained from this research will also serve as crucial recommendations for biomedical and fundamental research applications related to pathologies concerning the brain.

## 2 Materials and Methods

### 2.1 Materials

All N^α^-Fmoc-protected amino acids were purchased from GL Biochem Ltd. (Shanghai, China). Rink amide *p*-methylbenzhydrylamine (MBHA) resin, Fmoc-Lys(Mtt), piperidine, trifluoroacetic acid (TFA), pure oxyma, and 1-[bis(dimethylamino)methylene]-1H-1,2,3-triazolo[4,5-*b*]pyridinium 3-oxide hexafluorophosphate (HATU) were acquired from Iris-Biotec GmbH. 1,1,1,3,3,3-Hexafluoro-2-propanol (HFIP), nonadecanoic acid (C19), *N*,*N*’-diisopropylcarbodiimide (DIC), triisopropylsilane (TIS), matrix metalloproteinase-9 (MMP-9), Nile Red, thioflavin T, *N*,*N*-diisopropylethylamine (DIEA), temozolomide, 3-methyl-4-oxo-3,4-dihydroimidazo[5,1-*d*][1,2,3,5]tetrazine-8-carboxylic acid (temozolomide acid), 5(6)-carboxyfluorescein, 1-ethyl-3-(3-(dimethylamino)propyl)carbodiimide (EDC), 4-dimethylaminopyridine (DMAP), culture medium and its components, penicillin/streptomycin (P/S, 100 U/mL), l-glutamine, phosphate-buffered saline (PBS), and Lucifer Yellow assay were purchased from Merck (Milan, Italy). The slide chambers were purchased from Sarstedt. PrestoBlue assay and lactate dehydrogenase (LDH) assay were purchased from Thermo Fisher Scientific (USA). Annexin VI/PI was purchased from Elabscience (USA). Bioreactors and the peristaltic pump were acquired from IVTech (Italy). HBMEC and hPC-PL cells were purchased from Innoprot (Spain) and PromoCell (Germany), respectively.

### 2.2 Peptide Synthesis

Solid-phase peptide synthesis (SPPS) methodology was used for the synthesis of self-assembled peptides, as reported in Table 1.

**Table 1:** Peptide Sequence of Self-Assembled Peptides P1-P3, P2-D, P2-T, and P2-F

Peptide	Sequence
**P1**	GDDS-AAAAAA-K(C19)
**P2**	GKRS-AAAAAA-K(C19)
**P3** (delivery peptide)	HGLASTLTRWAHYNALIRAF-GKRS-AAAAAA-K(C19)
**P2-d** (peptide-bound drug)	TMZ-PLGSYL-SSS-GKRS-AAAAAA-K(C19)
**P2-t** (targeting peptide)	*falGea*-SSS-GKRS-AAAAAA-K(C19)
**P2-f** (labeled peptide)	FITC-PEG2-GKRS-AAAAAA-K(C19)

Fmoc-Lys (Mtt)-OH was chosen as the first amino acid for the incorporation of the lipid tail C19. The peptide synthesis was performed by using repeated cycles of Fmoc deprotection and coupling reactions. The Fmoc group was deprotected by treatment with a solution of 20% piperidine in DMF (2 × 5 min) under ultrasound,^30,31^ whereas each coupling reaction was achieved through two cycles. The first cycle was performed by treating the resin with a mixture of Fmoc-AA (3 equiv), HBTU (3 equiv), HOBt (3 equiv), and DIPEA (6 equiv) in DMF for 10 min under ultrasound. Then, the solution was discarded, and the reaction was repeated twice. At the end of the peptide elongation, the Mtt group was removed from the lysine side chain at the *C-*terminus by treatment with the mild acid cocktail TFA:TIS:DCM (1:5:94, v:v:v) through repeated cycles (10 times) for 25 min. The Mtt deprotection was monitored by the Kaiser test and high-performance liquid chromatography (HPLC) analysis after the acetylation test on a small amount of resin. After complete Mtt deprotection, the conjugation of nonadecanoic acid (2 equiv) was performed using HATU (2 equiv) and DIPEA (4 equiv) in NMP for 2 h under conventional stirring.

This coupling was repeated twice. After confirming the addition of C19 by HPLC and electrospray ionization mass spectrometry (ESI-MS) analysis, each self-assembled peptide was cleaved from the resin along with all protecting groups by treatment with the acid cocktail TFA:TIS: H_2_O (95:2.5:2.5, v:v:v) for 3 h under stirring. The resin was then filtered, and each peptide was precipitated with chilled diethyl ether (Et_2_O) and centrifuged at 6000 rpm for 15 min twice.

All crude peptides were purified by dissolving them in 1,1,1,3,3,3-hexafluoro-2-propanol (HFIP) (10%) and H_2_O (0.1% TFA). The peptide purification was performed by preparative HPLC on a Phenomenex Kinetex C18 column (5 μm, 100 Å, 150 × 21.2 mm) using linear gradients of MeCN (0.1% TFA) in water (0.1% TFA), from 10 to 90% over 35 min, with a flow rate of 15 mL/min and UV detection at 220 nm (see Figures S1–S6). The pure profile of each peptide was checked by analytical HPLC (Jasco LC-NetII/ADC) by using a Phenomenex Jupiter Proteo column (90 Å, 150 × 4.6 mm), and their identity was confirmed by ESI-MS analysis (Figures S7–S12).

### 2.3 Synthesis of Peptide-Bound TMZ

The drug TMZ was conjugated to the cleavage sequence “PLGSYL” which is recognized by the MMP-9 enzyme. The synthesis of the entire sequence and the addition of the lipid tail were performed as described previously. The drug TMZ was added as the final step at the *N-*terminus. After Fmoc deprotection, the coupling between 3-methyl-4-oxo-3,4-dihydroimidazo[5,1-*d*] [1,2,3,5]tetrazine-8-carboxylic acid (temozolomide acid) and the free amine was performed by using 1-ethyl-3-(3-(dimethylamino)propyl)carbodiimide (EDC, 2 equiv), 4-dimethylaminopyridine (DMAP 0.1 equiv), in DMF, overnight.^32^ The complete conjugation of TMZ at the N-terminus was confirmed by performing ESI-MS analysis. Then, the obtained P2-d was purified by HPLC as described above.

### 2.4 Fluorescein-Labeling of Peptide P2

5(6)-Carboxyfluorescein (FITC) was conjugated at the N-terminus of the peptide P2 to obtain P2-f (Table 1) and to perform the cellular uptake experiment. After the complete synthesis of the peptide P2 as described previously,^33^ Fam was attached at the N-terminus following deprotection of the Fmoc group. FITC (2 equiv) was coupled with COMU (2 equiv), Oxyma (2 equiv), and DIPEA (4 equiv) under stirring for 25 min. The coupling process was repeated twice. The Fam labeling was ascertained using HPLC and ESI-MS analyses, and then the peptide was purified as described above.

### 2.5 Peptide Assembly by Nile Red Assay

The critical aggregation concentration (CAC) of each self-assembled peptide, both alone and in combination with others, was calculated using Nile Red (NR) as the fluorophore.^34^ In this experiment, each peptide was dissolved in HFIP at a concentration of 400 μM, and then peptides were coassembled at specific ratios for final nanofiber concentrations of 0.8, 1, 5, 10, 15, 20, 30, 50, 100, 150, and 200 μM.^35^ The coassembled mixture composed of P1 + P2 + P3 + P2-t was tested at different ratios of P2-t. In particular, 1, 3, and 6 of P2-t were used, and the peptide molar ratios were 1:0.78:0.2:0.02 (ratio 1%), 1:0.74:0.2:0.06 (ratio 3%), and 1:0.68:0.2:0.12 (ratio 6%). In co-assembled mixtures composed of P1 + P2 + P3 + P2-t + P2-d (NF-TMZ), the peptide molar ratio was 1:0.54:0.2:0.06:0.2. Then, HFIP was removed under a nitrogen stream, reconstituted with water (500 μL), sonicated for 15 min, and freeze-dried. For the measurement, each sample was hydrated with a 500 nM NR solution in H_2_O for 1 h. The NR spectrum was recorded at a fluorescence emission range of 570–700 nm (slit width: 5 nm) and an excitation wavelength of 550 nm (slit width: 10 nm). When NR moves from water into aggregates, it produces a hyper-hypsochromic effect consisting of a blueshift and an increase in fluorescence intensity. For the CAC determination, each maximum emission fluorescence corresponding to the wavelength (*y*) was plotted as a function of the peptide concentration using the following sigmoidal Boltzmann equation:
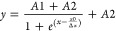


where *A*1 and *A*2 correspond to the upper and lower limits of the sigmoid, respectively. Instead, *x*0 and Δ*x* are the inflection point and steepness of the sigmoid, respectively.

### 2.6 Peptide Assembly by ThT Assay

Thioflavin T (ThT) is highly sensitive to hydrophobic environments and exhibits enhanced fluorescence at 482 nm upon binding with aggregated peptides.^36^ The ThT fluorescence intensity was measured at 25 °C after the formation of nanofibers using a Varian Cary Eclipse fluorescence spectrometer. The NFs were prepared and rehydrated with 200 μL of water after lyophilization for 1 h. Then, ThT at 25 μM was added to the nanofibers, and samples were excited at 450 nm (slit width: 5 nm) and fluorescence emission was recorded at 482 nm (slit width: 10 nm).^37^

### 2.7 Peptide Assembly by Electron Paramagnetic Resonance (EPR) Spectroscopy

The organization of the hydrophobic tails within the inner core of the nanofibers was investigated by EPR spectroscopy using two spin-labeled fatty acids, 5-DSA and 16-DSA, as spin probes. The samples analyzed by EPR were prepared by incorporating 1% of the spin probe into the fibers. An appropriate aliquot of a spin-probe solution in ethanol (1 mg mL^–1^) was added to the peptide mixture in HFIP before drying and reconstitution in water, yielding samples with nanofiber concentrations of 100 and 1 μM. 20 μM of these suspensions were inserted into glass capillaries and flame-sealed. These capillaries were placed in a standard 4 mm quartz EPR sample tube containing light silicone oil for thermal stability. EPR spectra were recorded on a 9 GHz Bruker Elexsys E500 spectrometer (Bruker, Rheinstetten, Germany) equipped with a super-high-sensitivity probehead. The temperature of the sample was controlled and kept constant at 25 °C during spectra acquisition by blowing thermostated nitrogen gas through a quartz dewar. The following instrument settings were used to perform measurements: sweep width, 140 G; resolution, 1024 points; modulation frequency, 100 kHz; modulation amplitude, 1.0 G; time constant, 20.48 ms; conversion time, 20.48 ms; incident power, 5.0 mW. 128 scans were accumulated to improve the signal-to-noise ratio. A quantitative analysis of each spectrum was performed by determining the values of the outer hyperfine splitting, 2*A*_max_, defined as the difference between the low-field maximum and the high-field minimum.^38^ This parameter is an empirical measure of the dynamics and order of the acyl chain segment bearing the paramagnetic label in self-assembled particles.^38^

### 2.8 Structural Characterization by Circular Dichroism (CD) Spectroscopy

The secondary structure and stability under different conditions were investigated by CD spectroscopy. The nanofibers NF-t and NF-TMZ were prepared to a final concentration of 40 μM as described above. After lyophilization and 1 hour of hydration necessary for nanofiber formation, CD spectra were recorded from 195 to 260 nm using a Jasco J-810 spectropolarimeter with a 1.0 cm quartz cell at room temperature.^39^ Each spectrum was obtained by averaging three scans and converting the signal to molar ellipticity. Moreover, the stability of NF-TMZ was studied under: i) the dilution effect, ii) different pH environments, and iii) the effect of ionic strength by varying the concentration of NaCl from 1 to 5 mM. For the evaluation of stability under the dilution effect, NF-TMZ was prepared at 50 μM and then diluted to concentrations of 40, 30, and 20 μM. For the pH environments and the effect of ionic strength, NF-TMZ was prepared at 40 μM and its stability was evaluated. Under all these conditions, each CD spectrum was recorded as described above.

### 2.9 Zeta Potential Measurements

Dynamic light scattering (DLS) measurements to determine the zeta potential of NF-t and NF-TMZ were conducted using a Zetasizer Nano-ZS (Malvern Instruments, Worcestershire, UK). The nanofibers were prepared as described above, and the analysis was performed with a He–Ne laser 4 mW operating at 633 nm at a scattering angle fixed at 173° and at 25 °C.

### 2.10 Morphological Characterization by Transmission Electron Microscopy (TEM)

Samples for transmission electron microscopy (TEM) imaging were prepared by dissolving the lyophilized fibers with milli-Q water at a 30 μM concentration and incubating them at room temperature for 3 h. This solution (5 μL) was deposited onto carbon-coated copper grids (Agar Scientific Ltd., product S160, 200 mesh) and allowed to dry at room temperature. Grids were glow-discharged at −15 mA for 1 min (PELCO easiGlow) before sample deposition. Grids were negatively stained with 5 μL of phosphotungstic acid (2% w/v, pH 7) solution for 2 min and air-dried. Micrographs were acquired in bright-field mode using a TEM TECNAI G2 20ST (FEI, Hillsboro, OR, USA), operating at an accelerating voltage of 120 kV. Nanofiber dimensions were measured using ImageJ software (National Institutes of Health, available free of charge at the website rsb.info.nih.gov/ij/), by calculating the average values based on at least 100 individual measurements.

### 2.11 Nanofiber Preparation for Biological Studies

All formulations (Table 2) for biological experiments were prepared at 100 μM. Each stock solution of the self-assembled peptide was prepared in HFIP, and they co-assembled with each other at the specific ratio defined. Then, the organic solvent was evaporated under a nitrogen stream; water (1 mL) was added, and each sample was freeze-dried. Nanofibers were allowed to form in water or in cell culture medium, and the hydration lasted 1 h just before the start of each biological experiment. The preparation of P2-f is reported above, and in order to achieve the desired concentration of FITC, peptide P2 was partially substituted with peptide P2-f to avoid other changes in the nanofiber composition.

**Table 2:** Nanofiber Formulation and Detailed Composition

Nanofiber	Composition
FITC-NF	P1 + P2 + P2-f + P3 (1:0.5:0.3:0.2)
NF	P1 + P2 + P3 (1:0.8:0.2)
NF (1% P2-t)	P1 + P2 + P3 + P2-t (1:0.78:0.2:0.02)
NF (3% P2-t) or NF-t	P1 + P2 + P3 + P2-t (1:0.74:0.2:0.06)
NF (6% P2-t)	P1 + P2 + P3 + P2-t (1:0.68:0.2:0.12)
NF-TMZ	P1 + P2 + P3 + P2-t + P2-d (1:0.54:0.2:0.06:0.2)
FITC-NF-t	P1 + P2 + P2-f + P3 + P2-t (1:0.44:0.3:0.2:0.06)
FITC-NF-TMZ	P1 + P2 + P2-f + P3 + P2-t + P2-d (1:0.24:0.3:0.2:0.06:0.2)

### 2.12 Cell Cultures

Glioblastoma/astrocytoma U-87 MG (U-87) and glioma U-118 MG (U-118) cell lines derived from the human brain were plated in 25 cm^2^ flasks. They were grown individually in Dulbecco’s modified eagle’s medium high glucose (DMEM), supplemented with fetal bovine serum (FBS, 10%), penicillin/streptomycin (P/S, 100 U/mL), and l-glutamine (L/GLUT, 2 mM, Sigma-Aldrich), in a humidified incubator (37 °C, 5% CO_2_). At 70% confluence, cells were individually detached with a 0.25% solution of trypsin-EDTA.

### 2.13 Set Up of Blood–Brain Barrier *In Vitro* Fluid Dynamic Model

The experiments were carried out using a Livebox2 (LB2), a millifluidic tool composed of a lower chamber separated by a porous membrane (ipPORE) from an upper chamber. These two chambers, with their independent flows, are connected to a peristaltic pump circuit (Liveflow).^26,27^ To mimic a reliable BBB *in vitro* dynamic model, we seeded different BBB cell components. HBMEC, isolated from a healthy human brain, were seeded on the porous membrane in the upper chamber of the LB2, on which human pericytes (hPC-PL) isolated from human placenta tissue, were also seeded on. Briefly, the porous membrane was first adapted in 80% ethanol for 15 min and then assembled into an LB2 between the upper and lower chambers. Later, a fibronectin coating was applied to the porous membrane, and after 24 h, HBMEC cells were seeded onto the membrane from the inlet tube of the upper chamber. The LB2 with its Liveflow was then placed in an incubator (37 °C/5% CO_2_) for 24 h. The circuit was first connected to the mixing chambers containing 8 mL of HBMEC complete medium, and then, the LB2 was connected to the Liveflow at a nominal flow of 250 μL/min. After 24 h, hPC-PL cells were seeded on the HBMEC cells on the porous membrane via the inlet tube of the upper chamber. The coculture was maintained for a week. After setting the flow conditions, 3D U-118 and 3D U-87 were transferred into the lower chamber of the LB2, which individually contained, in the upper chamber, the HBMEC and hPC-PL coculture for 24 hours, with the flow set at 120 μL/min.

### 2.14 Lucifer Yellow Assay

Lucifer yellow is a hydrophilic dye used to determine the permeability coefficient (Pc) of HBMEC and hPC-PL cultures. For different solutes, Pc values are 10^–4^ cm/min.^40^ To determine cell permeability translated into a spectral separation to determine fluorescence, the LY Stokes shift is 108 nm. On different days—1, 3, 6, and 8—we performed permeability measurements. Fluorescence was measured using a Bio-Tek Synergy HT Microplate Reader (Ex: 428 nm, Em: 536 nm). The permeation coefficient (Pc) was calculated using the following equation, as described elsewhere:^40^
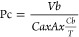
where *V*b is the volume of the lower chamber (1 mL), *C*b is the concentration of LY (μM) in the lower chamber, *C*a is the concentration of LY (μM) in the upper chamber, *A* is the membrane area (1.12 cm^2^), and *T* is the time of transport (3600 s). This test was useful in determining when HBMEC and hPC-PL cells form the tightest barrier.

### 2.15 Spectrofluorimetric Assay

The spectrofluorimetry assay was performed to evaluate the passage of FITC-NF. 20 μM of FITC-NF was injected into the upper chamber, corresponding to the membrane. A spectrofluorimetric experiment was performed on samples of medium taken at regular intervals (0.5, 1, 1.5, 2, 4, and 24 h). The supernatant (100 μL) was taken from the outlet tube of the upper chamber and the outlet tube of the lower chamber and placed in a 96-well plate. This solution was read by 491 to 516 nm fluorescence using a plate reader (Bio-Tek Synergy HT Microplate Reader).

### 2.16 Lactate Dehydrogenase (LDH) Assay

Lactate dehydrogenase (LDH) assay was used to test 3D U-87 and U-118 cytotoxicity in the lower chamber of LB2 after the passage of NF. LDH quantifies cellular cytotoxicity because damaged plasma membranes release lactate dehydrogenase, which converts lactate to pyruvate. H_2_O_2_ 1 mM was used as the positive control. To measure the absorbance, expressed in optical density (O.D.), a spectrophotometric reading was carried out at 490 nm using a plate reader (Bio-Tek Synergy HT Microplate Reader). Three assays were performed, and for each experimental class, the test was performed in triplicate.

### 2.17 Cell Uptake Evaluation

To evaluate the cell uptake of nanofibers, FITC-NF decorated with different percentages of P2-t (1-3-6%) or FITC-NF-TMZ was incubated with U-118 and U-87 cells grown on slide chambers (Sarstedt). Cells were seeded in chamber slides at a density of 25,000 cells/chamber and were treated with each formulation (Table 2), where the concentration of FITC corresponded to 9 μM. After 3 h, cells were fixed with 4% paraformaldehyde for 15 min, washed in 0.1 M PBS, counterstained with Hoechst (5 μg/mL)), and then, after mounting with IBIDI aqueous mounting medium, observed under an Axioskop microscope (Zeiss) and acquired by Axiovision software (Zeiss). Image analysis was performed by Zen (Zeiss) and Fiji software. Cells were analyzed by fluorescence microscopy to evaluate fiber cell uptake and cell morphology after treatments. Standardized fluorescence measurements were obtained by spotting different dilutions of FITC-NF-t or FITC-NF-TMZ on slides and acquiring images under the same conditions as those used for the treated cells, as described elsewhere.^12^ Once the appropriate percentage of the target peptide (3%) was evaluated, we carried out U-118 and U-87 cell uptake experiments at the appropriate percentage.

### 2.18 2D and 3D Cell Treatment and Viability Assay

For 2D experiments, U-118 and U-87 cells were seeded in 48-well plates, at approximately 20,000 cells per well in DMEM culture medium with 10% FBS, under standard cell culture conditions (5% CO_2_, 37 °C). Once the cells were attached, they were treated for 24, 48, and 72 h with 1) NF-TMZ carrying TMZ at different concentrations: 2.5, 5, 7.5, and 10 μM; 2) NF-t; 3) free TMZ at different concentrations (0, 10, 100, and 250 μM). TMZ was previously dissolved in dimethyl sulfoxide (DMSO) at a stock concentration of 0.133 M and then diluted with culture medium according to the concentrations reported in the literature.^41^ Control experiments were performed in the presence of DMSO (250 μM), which corresponded to the highest TMZ concentration. At the end of the incubation time, a cell viability assay was performed using the PrestoBlue assay: the cells were incubated for 1 h according to the manufacturer’s protocol, and then absorbance was measured at 570 nm (with 600 nm as the reference wavelength) using a Bio-Tek Synergy HT Microplate reader.

Once the appropriate concentration corresponding to the greatest lowering of cell viability was established, we proceeded with the 3D U-118 and 3D U-87 cell assays. To produce 3D U-118 and 3D U-87 cells, we used the hanging drop method. Briefly, 10,000 cells per drop were placed in the lid of a 100 mm dish, creating a hydration chamber by placing PBS in the dish bottom. After incubating for 48 h at 37 °C/5% CO_2_, the aggregates were transferred to a 48-well plate (5 spheroids per well: approximately 50,000 cells/well). The 3D cells were treated every 24 h for 3 days. Each treatment was conducted on 10 spheroids: approximately 100,000 cells for each experimental class: 1) control; 2) NF-t; 3) NF-TMZ (10 μM); 4) free TMZ (10 μM). At the end of the incubation time, a cell viability assay was performed with using the PrestoBlue assay: the cells were incubated for 90 min, according to the manufacturer’s protocol. At the end of the incubation time, we measured the absorbance at 570 nm (with 600 nm as the reference wavelength) using a Bio-Tek Synergy HT Microplate reader.

To verify whether NF-t or NF-TMZ influenced healthy brain endothelial cells constitutively expressing EGFRs, the experiment described above was conducted on human brain microvascular endothelial cells (HBMEC) and brain endothelial cells (bEnd.3) to assess their effects . Cells were seeded in a 96-well plate (15,000 cells per well) in their appropriate culture medium. After 24 h, cells were treated likewise with U-118 and U-87 cells with 10 μM TMZ, both conjugated and unconjugated with NF-t. The control was performed on cells cultured only in the medium. Under the same conditions as for 2D cells, the PrestoBlue assay was performed. Since HBMEC and bEnd.3 cells do not overexpress metalloproteinases, TMZ release from NF-TMZ was induced by adding matrix metalloproteinase-9 (MMP-9) to the incubation medium. TMZ release from NF-TMZ was evaluated using matrix metalloproteinase-9 (MMP-9). In particular, NF-TMZ was prepared as reported before and hydrated in the following buffer solution: 50 mM HEPES, 200 mM NaCl, 10 mM CaCl_2_, and 1 mM ZnCl_2_, at pH 7. The MMP-9 was preactivated with APMA 100 μM and Tris-HCl 50 mM (pH 7.2) and was left at 37 °C for 3 h.^33,35^ Once activated, MMP-9 was added to NF-TMZ and the culture medium at a final concentration of 40 nM; and cells were incubated for 72 h.

### 2.19 TMZ Release by Enzymatic Cleavage

TMZ release from our nanofibers by the proteolytic cut of the enzyme MMP-9 was also evaluated using UV–visible spectroscopy.^35^ A nanofiber composed of P1 + P2-d (1:1) was prepared at a final concentration of 800 μM and then diluted with an MMP-9 solution to achieve a P2-t’s concentration of 200 μM, suitable for UV measurements. The MMP-9 used for cleavage was preactivated with 100 μM APMA and 50 mM Tris-HCl (pH 7.2) and left at 37 °C for 3 h. The nanofiber was hydrated for 1 h with buffer solution: 50 mM HEPES, 200 mM NaCl, 10 mM CaCl_2_, and 1 mM ZnCl_2_, at pH 7, and then was incubated with MMP-9 (40 nM) at 37 °C. At each time point (1 and 3 h), 50 μL was taken from the mixture, centrifuged at 13,000 rpm for 30 min, and the supernatant was analyzed by UV/vis spectroscopy (NanoDrop 2000/2000C, Jasco, Milan, Italy) following absorbance at 329 nm (TMZ).

### 2.20 Annexin V-FITC/Propidium Iodide Assay

To identify apoptotic and necrotic cells, Annexin V-FITC/Propidium Iodide (PI) assay was performed according to the manufacturer’s protocol. Annexin V highlights apoptotic cells by binding to phosphatidylserine (PS) on their membranes . Propidium Iodide (PI) binds to the DNA of necrotic cells, as it is not permeable to the cell membrane. Cells lose the membrane integrity, Annexin V-FITC/PI can enter the cells. After the 3D cell treatment, cells were stained with Annexin V-FITC/PI for 20 min in the dark. After washing with PBS, nuclei were labeled with 4′,6-diamidino-2-phenylindole (DAPI) for 5 min. Images of the 3D cells were acquired using the JuLi Stage_RealTime Cell History Recorder microscope with a 10x objective, using different channels: RFP, GFP, and DAPI. The images were corrected for brightness and contrast using Fiji software and analyzed to evaluate morphological parameters after treatments. For each experimental condition, three different assays were repeated.

### 2.21 Spectrofluorimetry Assay of FITC-NF-TMZ and FITC-NF-t

A spectrofluorimetry assay was also performed to evaluate the passage of FITC-NF-TMZ (10 μM) compared to FITC-NF-t. The compounds were injected into the upper chamber, corresponding to the membrane where HBMEC and hPC-PL coculture are seeded. A spectrofluorimetric experiment was performed on samples of medium taken at regular intervals (0.5, 1, 1.5, 2, 4, 24 h). The supernatant (100 μL) was taken from the outlet tube of the upper chamber and from the outlet tube of the lower chamber and placed in a 96-well plate. These solutions were read at 491–516 nm fluorescence using a plate reader (Bio-Tek Synergy HT Microplate Reader).

### 2.22 Cell Viability Assay on 3D Dynamic *In Vitro* BBB Model

To evaluate the effects of NF-TMZ on fluid-dynamically cultured 3D GBM cells, we set up different bioreactors containing HBMEC and hPC-PL cells in the upper chamber and 3D U-118 cells or 3D U-87 cells in the lower chamber. NF-TMZ (10 μM), NF-t, and free TMZ (10 μM) were injected into the upper chamber, and their effects were evaluated after 72 h using the PrestoBlue viability assay. Briefly, 72 h after treatment, spheroids in the lower chamber were taken out and placed inside 48-well plates, and the PrestoBlue viability reagent (1:10) was added for 1.5 h. The absorbance was quantified using a Bio-Tek Synergy HT Microplate Reader. The control bioreactor was performed with HBMEC and hPC–PL cells in the upper chamber; and 3D U-118 MG cells and 3D U-87 MG cells in the lower chamber without treatment.

### 2.23 Statistical Analyses

All experiments were performed in triplicate and expressed as means ± SEM. For 2D U-118 and U-87 experiments, statistical significance between groups was assessed by a one-way ANOVA, with Bonferroni’s multiple comparisons posttest. For 3D cell experiments, statistical analysis was performed through the analysis of variance (ANOVA) followed by Dunnett’s posttest. The two-tailed Mann–Whitney test was performed to evaluate treated groups, compared with the appropriate control group. As for Annexin/PI test, statistical significance was evaluated running Kruskal–Wallis nonparametric test with Dunn’s comparison as posttest. Data were considered statistically significant for: *p < 0.05, **p < 0.01, ***p < 0.001, ****p < 0.0001. All graphs were obtained using the program Origin (Origin 2003. Origin 7.5. OriginLab Corp., Northampton, MA).

## 3 Results

### 3.1 Nanoplatform Design and Synthesis

Self-assembling peptides possess unique physicochemical properties—including biocompatibility, biodegradability, and the ability to tune size, shape, and surface area—which make them highly suitable for drug delivery applications.^42^ Herein, we present nanofibers (NFs) designed and engineered for TMZ delivery, building on our previous studies on the use of self-assembling PAs as building blocks for the construction of supramolecular platforms.^33,35^ Self-assembling PAs were conceived with both hydrophilic and hydrophobic domains covalently linked to each other, enabling precise control over their self-aggregation behavior. This structural arrangement enables precise control over the aggregation process, allowing the formation of materials with tunable properties. The hydrophobic domain includes a hexa-alanine sequence and an alkyl chain with 19 carbon atoms (C19) linked to the ε-amino group of the lysine side chain at the C-terminus.^33,35^ Regarding the hydrophilic domain, negatively charged residues (Asp) and positively charged residues (Lys, Arg) were chosen since they are involved in electrostatic interactions for the assembly into supramolecular structures. Each peptide—P1, P2, P3, P2-t, P2-d, and P2-f—used in our study featured the same hydrophobic domain, while the hydrophilic domain, necessary to improve peptide solubility, changes to keep a 1:1 ratio between negative and positive amino acids for nanofiber formation. Specifically, P1 has a hydrophilic domain made of aspartic acid as negatively charged residues, while P2, P3, P2-t, P2-d, and P2-f have a positively charged domains These PAs spontaneously form supramolecular nanofibers upon immersion in an aqueous solution, with the key advantage that targeting and delivery moieties, along with the drug, can be directly located on the surface of the nanosystem.

**Figure 1:**
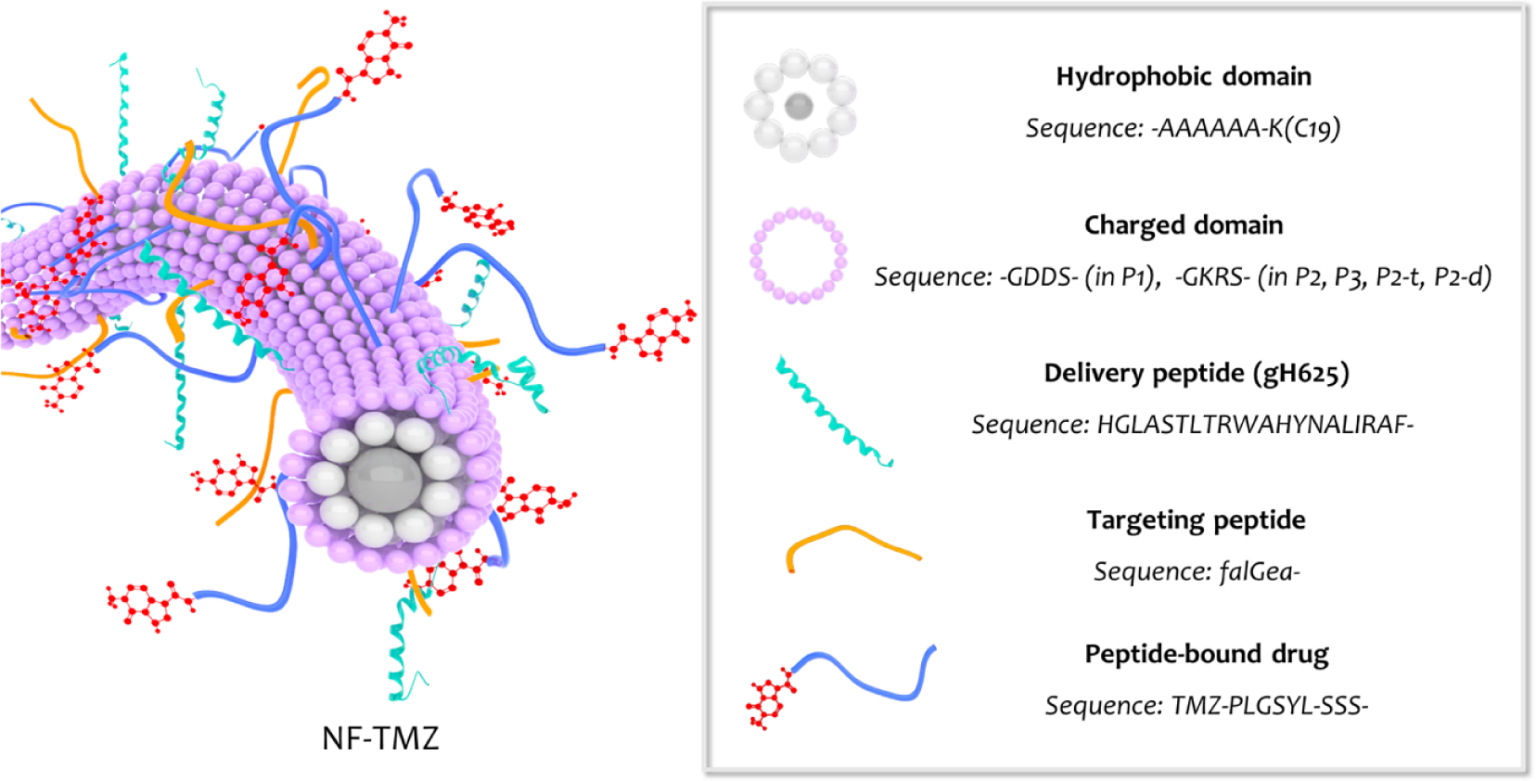
Schematic representation of NF-TMZ with biological entities covering its surface.

In our nanofiber design, each self-assembled peptide has a specific structural or biological role (Figure 1): i) P1 and P2 constitute the nanofiber structure with the hydrophobic domain forming the NF core, while the hydrophilic domain stabilizes the formation of the nanofiber and improves its solubility; ii) the peptide P3 includes the sequence of the cell-penetrating peptide gH625 that is able to cross the BBB as demonstrated in our previous studies;^25,26^ iii) the peptide P2-t includes the targeting peptide *falGea* showing a high binding affinity to EGFR and mutation variant III (EGFRvIII) overexpressed on neovasculature, vasculogenic mimicry, and tumor cells;^28^ iv) the peptide P2-d carries the drug TMZ that is intended to be released by the proteolytic cut of MMP-9 overexpressed at the tumor site. Indeed, it has been proven that GBM cells produce large amounts of MMP-9, as a regulator of the tumor microenvironment and are specifically required for cancer development, progression, invasion, and metastasis formation;^29^ v) the peptide P2-f carries the labeling group and is used for microscopy studies.

All these peptides participate in the nanofiber assembly and formation, the surface of which resulted in being decorated by the biological players CPP, TM, and drug to achieve selective and targeted drug release at the glioblastoma site. When peptides are mixed in solution below their critical aggregation concentration (CAC), the formation of nanofibers is not observed; in contrast, when the peptides coassemble above the CAC in solution, the hydrophobic and hydrophilic domains interact and favor nanofiber formation.

Moreover, regarding their synthesis, all peptides were synthesized via solid-phase peptide synthesis by using the Fmoc/tBu strategy. The long lipid tail (C19) was conjugated to the lysine side chain added at the C-terminus of each peptide. The lysine protected with the Mtt group in its side chain was chosen as the lipidation site because the Mtt group can be removed under mild acidic conditions, which easily allows the conjugation of the lipid C19. Instead, TMZ was covalently coupled after the cut sequence (PLGSYL), which should be recognized by MMP-9 following an on-demand approach. Finally, all PAs were cleaved from the resin, purified by HPLC, and then characterized by ESI-MS.

### 3.2 Nanofiber Formulation and Crossing of the BBB

Our previous studies demonstrated a strong tendency for each peptide—P1, P2, and P3—to self-assemble both individually and in combination with one another.^33,35^ When P1, P2, and P3 were mixed in solution at a specific molar ratio of 1:0.95:0.05, a value of the CAC of 15.2 ± 0.9 μM was calculated, and the formation of nanofibers with a diameter of ca. 12 ± 2 nm and a length of ca. 150 ± 50 nm was observed by TEM analysis.^23^ The equimolar ratio of negatively and positively charged peptides leads to the irreversible formation of catanionic aggregates. Indeed, once formed, nanofibers can be diluted without disassembly, as previously demonstrated by CD studies.^33,35^ The EPR characterization of the inner core formed by the C19 acyl chains indicates a compact molecular organization that strongly hinders rotational mobility. It is interesting to note that the 2*A*_max_ value observed for 5-DSA (85 G) is higher than that observed for acyl chains in any type of lipid self-assembly (see Figure S13). Another peculiarity of the organization of the acyl chains in our nanosystem is the absence of the mobility increase usually observed from the more external lipid segments toward the core center: a similar 2*A*_max_ value is observed for 16-DSA (81 G). Moreover, in cellular uptake studies, this nanofiber decorated with the peptide gH625 (peptide P3) showed a significant cellular internalization and nuclear delivery of the drug in triple-negative breast cancer cell lines.^33,35^

**Figure 2:**
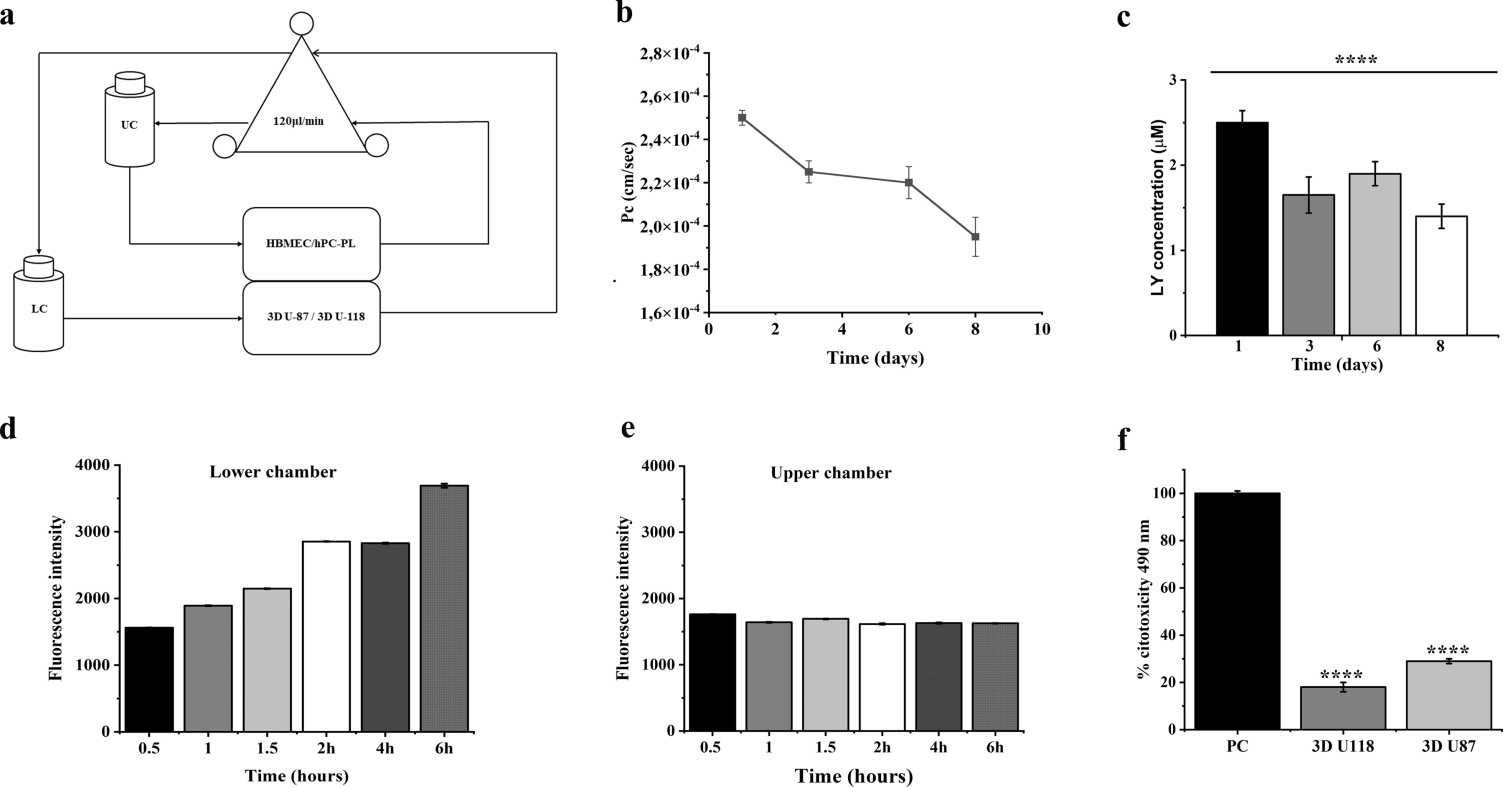
Panel a: Set up of the BBB *in vitro* fluid dynamic model. In the Livebox2 bioreactor, HBMEC are in coculture with hPC-PL in the upper chamber on the porous membrane. This chamber was connected to the upper reservoir (UR) and to a peristaltic pump. In the lower chamber, 3D U-87 and 3D U-118 individually are seeded and connected to the lower reservoir (LR) and to a peristaltic pump. Panel b: Luciferase yellow permeation through HBMEC and hPC-PL bilayer cells. Panel c: Luciferase yellow concentration (μM) in 8 days. The graph shows the means ± SEM of three experiments. Panels d and e show the spectrofluorometric results of FITC-NF delivery across a BBB dynamic *in vitro* model obtained for the lower chamber (panel d) and for the upper chamber (panel e). Panel f shows LDH assay on 3D U-87 or 3D U-118 cells seeded in the lower chamber of the LB2 BBB dynamic model H_2_O_2_ (PC) was used as a positive control. The graphs represent the means ± SEM of three experiments.

Thus, starting from this proof of concept, the same nanofiber (NF, composition: P1 + P2 + P3, 1:0.8:0.2) with the same molar ratio of peptide P3 was used to evaluate its crossing through the BBB in a biodynamic reactor model. In particular, we have reconstructed a BBB *in vitro* dynamic model using a bioreactor with double flow independent chambers: in the upper chamber, HBMEC, isolated from the healthy human brain, is seeded on a porous membrane, and above the HBMEC, there is hPC-PL, isolated from human placenta tissue; the lower chamber of the bioreactor contains spheroids made of 3D U-87 or 3D U-118 cell lines from the human brain. First, a Lucifer Yellow (LY) assay was performed to evaluate and confirm the barrier’s functionality. LY was added to the upper chamber of LB2 (Figure 2, panel a); higher concentrations of LY indicate that the barrier is immature, while lower concentrations indicate a functional barrier. LY permeability decreases until day four and then remains constant until day six to finally decrease up to day eight (Figure 2, panels b, c). The data clearly point to the obtainment of a functional barrier, warranting the reliability of all downstream experiments.

To evaluate the passage across the BBB of FITC-NF (composition: P1, P2, P2-f, P3, 1:0.5:0.3:0.2), we used the LB2 bioreactor containing HBMEC and hPC-PL in the upper chamber and 3D U-87 or 3D U-118 in the lower one. Medium samples were taken from both the outlet and inlet of the camera (upper and lower) at different time points of 0.5, 1, 1.5, 2, 4, and 24 h. We observed different trends between the upper and lower chambers. An increase in FITC-NF fluorescence was observed in the lower chamber compared to the upper one **(**Figure 2, panel d). Indeed, in the upper chamber, we found a continuous increase in fluorescence, whereas, in the lower chamber, the fluorescence remained constant starting from 0.5 to 24 h without a substantial increase (Figure 2, panel e).

We also determined the cytotoxicity of the NF on 3D U-87 or 3D U-118 cells located in the lower chamber. The graph clearly showed lower cytotoxicity for 3D cells compared to the positive control (PC, 1 mM H_2_O_2_). This indicates that the nanofiber is not toxic to the spheroids (3D U-87 and 3D U-118) and can be effectively used to bind, on demand, the drug (TMZ) and to evaluate its effects on the 3D cells (Figure 2, panel f).

### 3.3 EGFR-Targeting: NFs Formulation and Characterization

Once the ability of NFs to cross the BBB was confirmed, we aimed to achieve the selective TMZ release at the GBM site; thus, we prepared several NFs decorated with three different percentages of the peptide P2-t, which is able to effectively bind to the receptors EGFR and EGFRvIII overexpressed on GBM cells.

**Figure 3:**
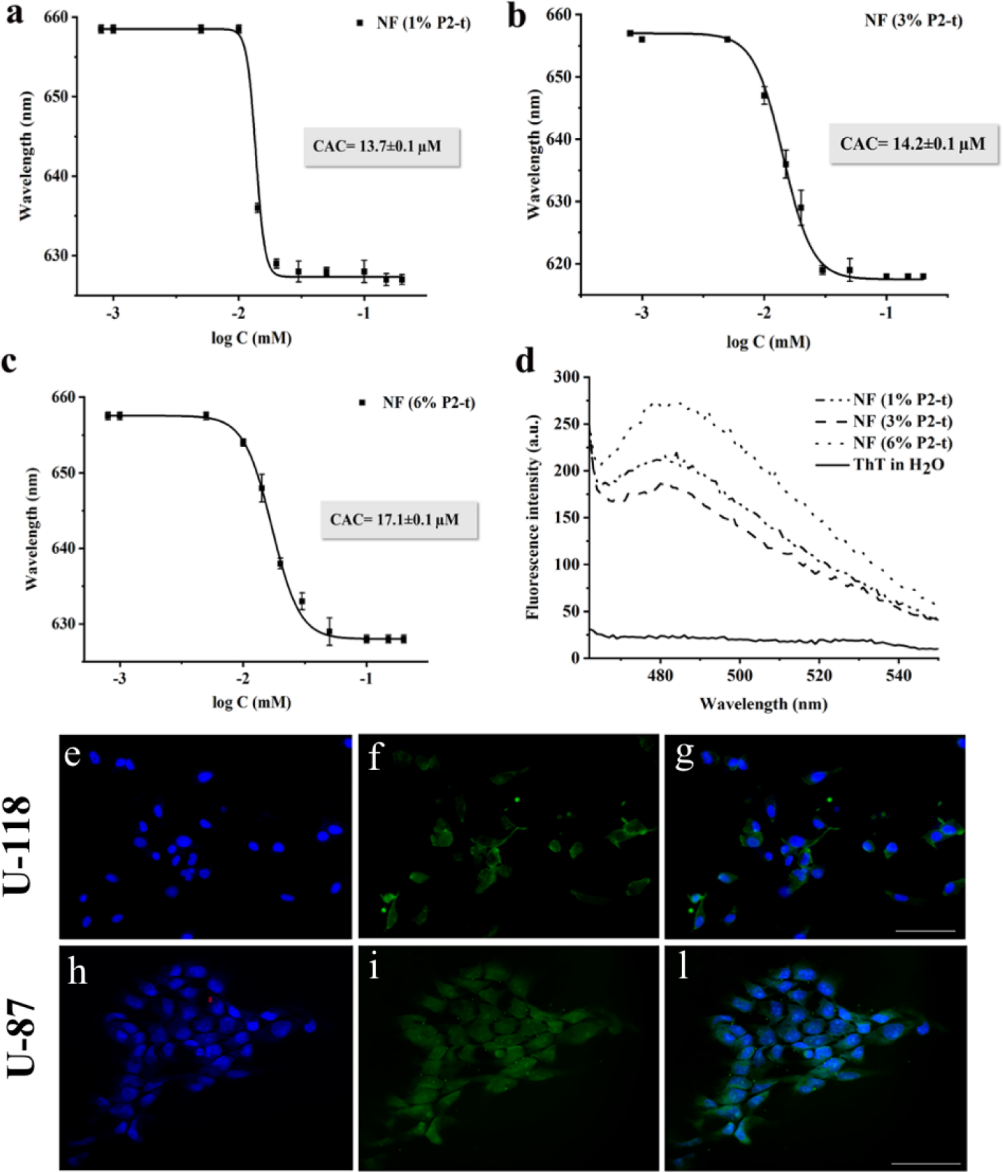
CAC for NFs with different percentages of P2-t (panels a, b, and c). ThT spectra recorded for NFs with 1, 3, and 6% of P2-t (panel d). Representative images of the FITC-NF-t uptake by U-118 and U-87 cells. The NF-t green fluorescence can be seen in the cytoplasm of both cell lines (f,i); e,h: cell nuclei colored in blue (Hoechst); g,l: merge of blue and green channels. Bars = 50 μm.

The percentages of 1, 3, and 6% were used to decorate the nanofiber. First, we verified the formation of the nanofibers through the calculation of the CAC values for the coassembled nanofibers while keeping an equal ratio of positive/negative charges, using the NR-based fluorescence assay. The decreased polarity of the hydrophobic environment produces a hyper-hypsochromic effect (blueshift and intensity increase) on the NR fluorescence in comparison with its fluorescence recorded in an aqueous solution. Regardless of the concentration, the addition of the peptide P2-t did not induce significant modifications in the CAC values. In fact, CAC values are very similar in the presence of 1, 3, and 6% of peptide P2-t, as reported in Figure 3 (panels a–c). In addition, the peptide aggregation and nanofiber formation were monitored by the Thioflavin T (ThT) assay. ThT is widely used to analyze the formation of self-assembled nanostructures; in fact, the interaction within these structures impedes rotation between the benzene and benzothiazole rings, which results in enhanced fluorescence. As observed in Figure 3 panel d, an increase in ThT fluorescence intensity at 482 nm was recorded with increasing concentrations of the peptide P2-t on the NF surface.

The obtained biophysical results did not indicate a different behavior among these three distinct nanofiber preparations in terms of peptide assembly and nanofiber formation; therefore, the appropriate percentage of P2-t was selected through fluorescence microscopy by performing the cellular uptake experiment on GBM cells (Figure 3, panels e-f-g-h-i-l). This experiment was conducted on nanofibers carrying 1, 3, and 6% of the targeting peptide (P2-t) on the surface (see Figure S14). The analysis of all obtained images prompted us to select nanofibers carrying 3% of P2-t (hereafter referred to as NF-t) for subsequent cell uptake experiments. In particular, as all the moieties are located on the surface of the nanofiber, it is important to avoid any overcrowding, which could eventually cause further aggregation among nanofibers and shielding of the active groups. Thus, we decided to use the lowest percentage, providing a good uptake in both cell lines. We reported in Figure 3 (panels e-f-g-h-i-l) the uptake of the fiber carrying 3% of P2-t (NF-t) with 2D U-118 and 2D U-87 cells; the images clearly confirmed that uptake occurred in both cell types.

### 3.4 Nanofiber Bearing the Drug (TMZ): Biophysical and Structural Characterization

Once the percentage of P2-t to add on the NF surface was determined, TMZ covalently bound to peptide P2 (P2-d) was added at a percentage of 10% to obtain NF-TMZ (composition, P1:P2:P3:P2-t:P2-d, 1:0.54:0.2:0.06:0.2). Its assembly was characterized in terms of aggregation and structural stability in different environments. A percentage of 10% of TMZ was selected for the biological experiments; in fact, this concentration corresponds to 10 μM TMZ which is 10 times lower than the concentration reported as the active concentration in the literature.^41^ We decided to use such a low concentration to demonstrate that the NF-t can effectively deliver TMZ to its target site, and a lower dose is able to produce the same biological effect.

**Figure 4:**
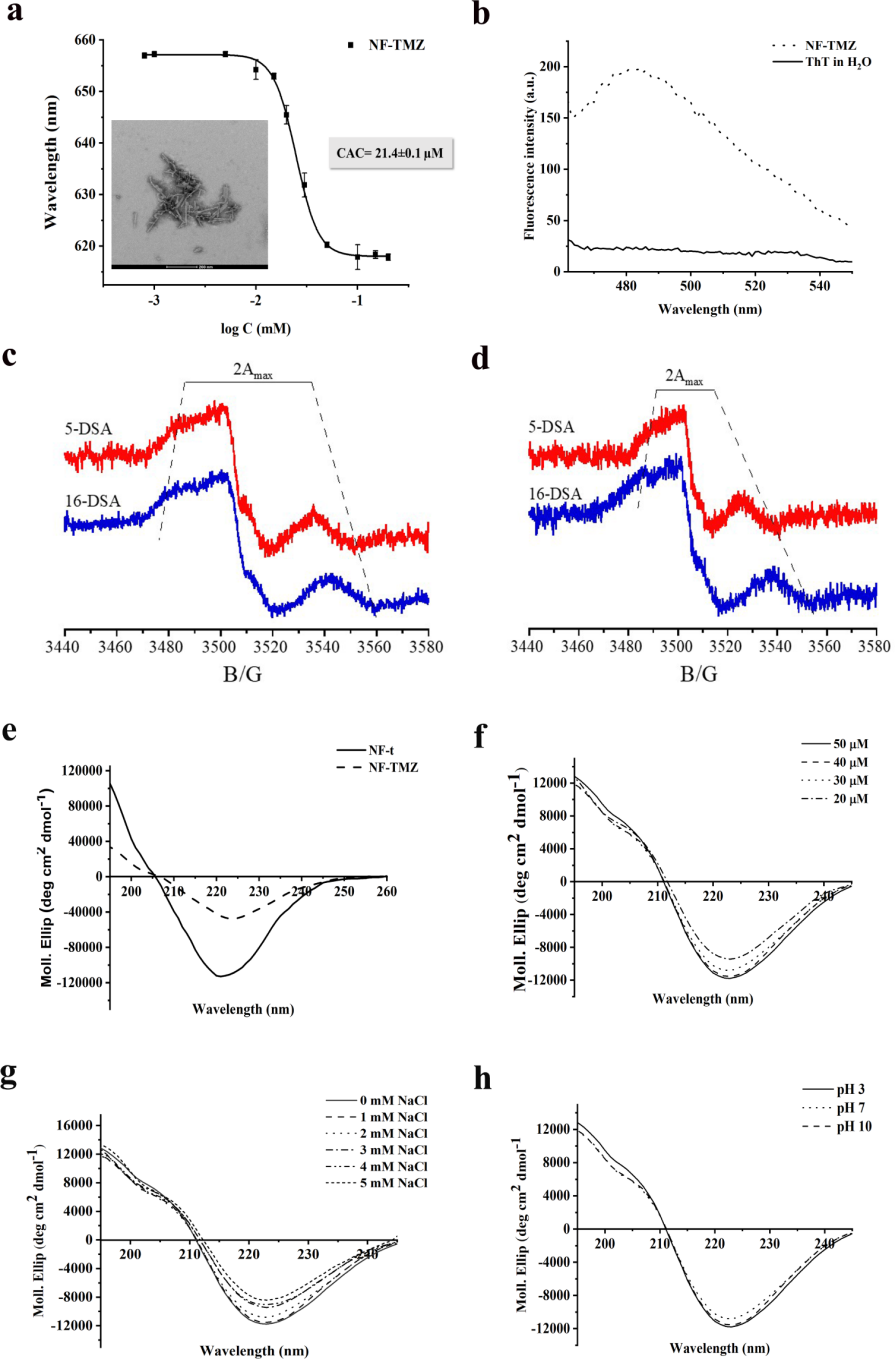
Panels a, b report the CAC value and the ThT spectrum of NF-TMZ, respectively. Panels c and d report 2*A*_max_ values recorded for NF-t (c) and NF-TMZ (d). Panel e shows CD spectra of NF-t and NF-TMZ at the concentration of 40 μM. Panels f, g, and h show the CD spectra of NF-TMZ under different conditions: (f) dilution effect; (g) ionic strength; (h) pH 3, 7, and 10.

By performing the NR assay, we calculated a CAC value of 21.4 ± 0.1 μM for NF-TMZ bearing all biological entities (P3, P2-d, P2-t), indicating a slightly lower tendency to form fibers in the presence of TMZ on the surface compared to the nanofiber (NF-t) decorated solely with P3 and P2-t (Figure 4, panel a). The peptide aggregation and the formation of the nanofiber were also supported and confirmed by the significant increase in the fluorescence intensity of ThT around 482 nm (Figure 4, panel b). Moreover, the analysis of the TEM images confirmed the formation of nanofibers with a length of 160 ± 40 nm and a diameter of 11 ± 3 nm (Figure 4, panel a, and Figure S15).

Furthermore, the analysis of the zeta potential indicated that the addition of the drug (P2-d) did not induce a variation in the surface charge which could influence the colloidal stability and the uptake of the nanofiber, since we measured a positive value for both NF-t (+4.4 ± 1.2 mV) and NF-TMZ (+7.5 ± 0.4 mV). The slight increase in the zeta potential after the addition of TMZ to the nanofiber surface clearly confirmed the presence of TMZ on its surface.

Interestingly, the EPR experiments showed a significant difference between NF-t and NF-TMZ with regard to the nanofiber core formed by acyl chains. Specifically, for NF-t, without TMZ on its surface, a slightly increased mobility of the acyl chains in the nanofiber core is due to the inclusion of P2-t in their formulation; this effect is more evident for the outer chain segments than for the inner ones. The 2*A***_max_** values observed for 5- and 16-DSA are 65 and 76 G, respectively (Figure 4, panel c). This evidence suggests that the steric hindrance between the bulky P2-t headgroups induces a less ordered packing of the acyl chains. Interestingly, when we added P2-d to the nanofiber composition (NF-TMZ), it further enhanced the effect on the acyl chain organization observed for P2-d, as highlighted by the EPR results (Figure 4, panel d). The 2*A*_max_ values further decrease (46 and 66 G for 5- and 16-DSA, respectively), indicating a significant effect of the steric hindrance of the drug exposed on the fiber surface.

Furthermore, the addition of TMZ to the nanofiber surface did not cause a structural change, as evidenced by the circular dichroism (CD) analysis reported in Figure 4. The CD spectrum of NF-t showed a β-type conformation featured by a negative band at ∼220 nm, and the same conformation was preserved when we added P2-d to NF-t (Figure 4, panel e) but a change in the aggregation was observed. Indeed, the CD spectrum of NF-TMZ indicated highly aggregated β-structures, since the CD intensity was lower than that recorded for NF-t without TMZ at the same concentration of 40 μM.

Before evaluating the biological effectiveness related to TMZ delivery by NF-TMZ, we investigated the stability of NF-TMZ under different conditions, including the dilution effect, change in ionic strength, and pH environments. The CD analysis (Figure 4, panel f) showed that NF-TMZ was stable under the dilution effect and preserved its β-type conformation at concentrations of 50, 40, 30, and 20 μM, as evidenced by the sufficient overlapping of all CD spectra. Similarly, NF-TMZ appeared to be stable under different concentrations of NaCl ranging from 1 to 5 mM and three different pH conditions (3, 7, and 10), conserving the β-type conformation with the minimum at 220 nm (Figure 4, panels g and h).

**Figure 5:**
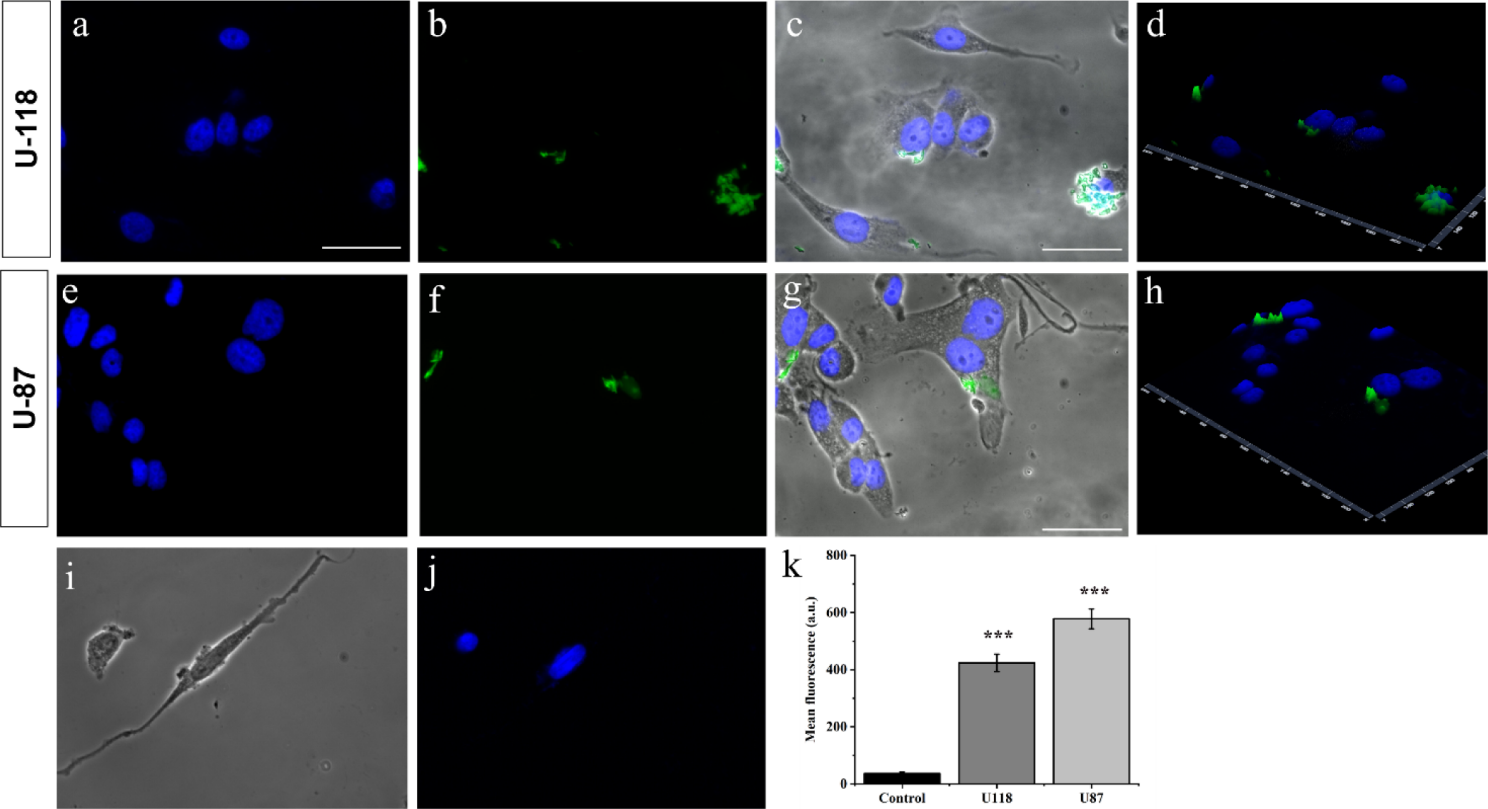
Panels a–j report representative images of U-118 and U-87 cells treated with FITC-NF-TMZ. Scale bar 50 μm. (a–j) Nuclei stained with Hoechst; (b,f) green channel showing fiber; (c,g) blue, green, and bright channels merge showing specific accumulation of fiber inside cells; (d,h) 2.5 D reconstruction of the blue and green channels showing specific localization of signals and their reciprocal proximity; (i,j) negative control (without FITC-NF-TMZ). Panel k reports mean cell fluorescence in 2D U-118 and U-87 cells treated with FITC-NF-TMZ; control is represented by cells not treated with FITC-NF-TMZ. Data ± SEM are reported. Unpaired *t* test, two tailed value ***p < 0.001.

Furthermore, we observed that the addition of TMZ to the nanofiber surface did not influence its uptake, attributed to the presence of the peptides P2-t and P3 on U-118 (Figure 5, panels a–d) and U-87 cells (Figure 5, panels e–h). Specifically, after 3 h from treatment, the FITC-NF-TMZ enters 2D U-118 and U-87 cells and can be easily detected in the cytoplasm. We observed that the two cell lines show different efficiencies of internalization with a different amount of uptake, although in both cases the nanofibers accumulate in the area surrounding the cell nuclei (Figure 5, panels c, g). To confirm our analysis, we evaluated the mean cell green fluorescence, as shown in Figure 5, panel k; the green fluorescence is greatly increased compared with negative controls, indicating that the nanofiber is located inside the cell, and we also observed greater internalization in U-87 cells compared to U-118.

### 3.5 TMZ Release by Proteolytic Cut of MMP-9

TMZ release from the nanofibers was evaluated by using UV–vis spectroscopy. The nanofiber decorated with P2-t at a concentration of 200 μM was incubated with the enzyme MMP-9 (40 nM) at 37 °C for 1 and 3 h. At each time point, 50 μL of the mixture was taken and centrifuged to eliminate the nanofiber, and then the supernatant was analyzed following the absorbance at 329 nm. The results showed a rapid release of TMZ around 68 ± 1% after 1 h and 80 ± 2% after 3 h of incubation (Table 3). Moreover, the TMZ release was also evaluated and confirmed in *in vitro* experiments performed on the brain endothelial cell line bEnd.3.

**Table 3:** Percentage of TMZ Released after the Incubation with MMP-9

Time (hours)	TMZ release (%)
1	68 ± 1
3	80 ± 2

### 3.6 Cell Viability Studies in 2D and 3D Experiments

**Figure 6:**
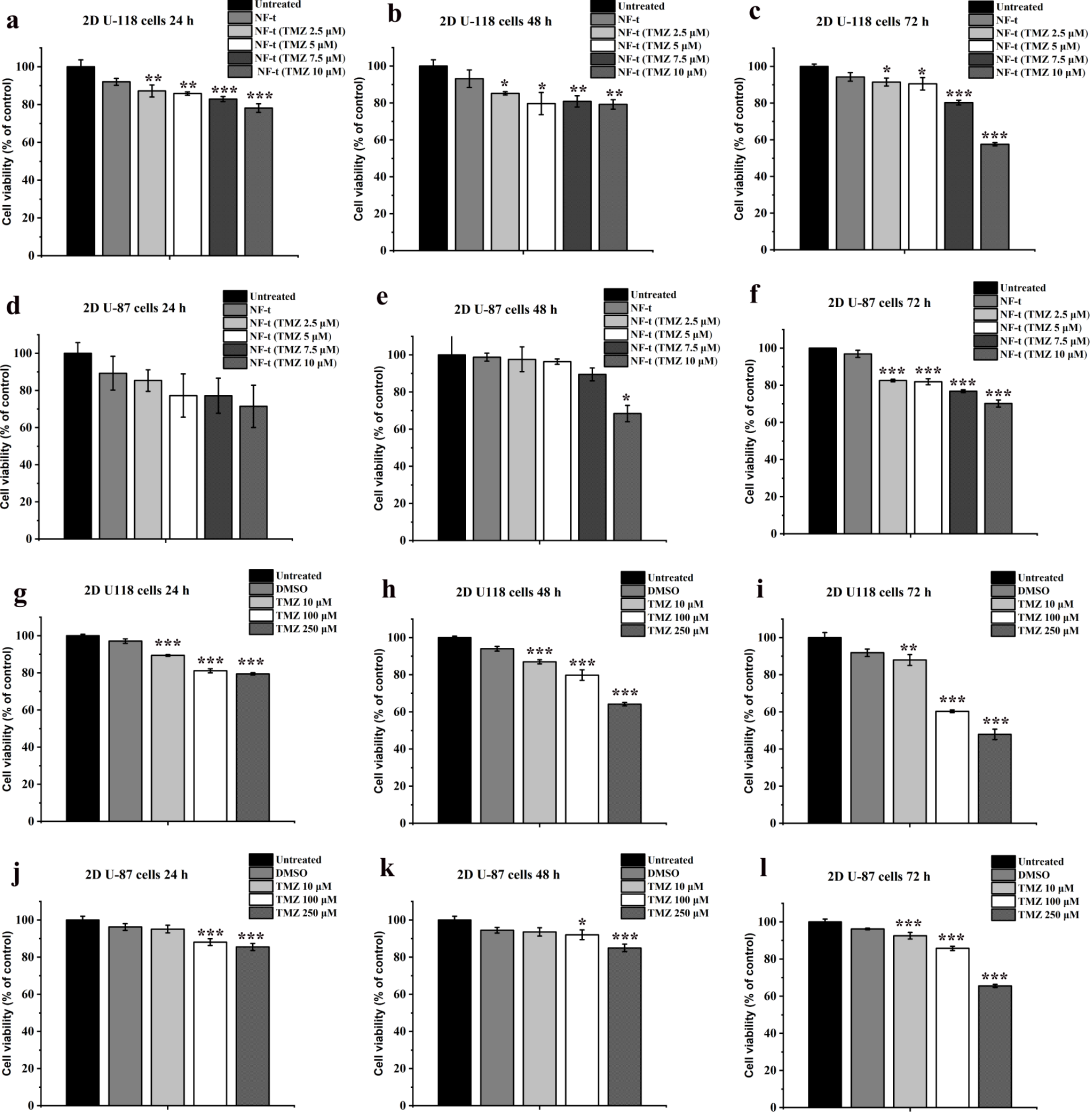
2D U-118 (panels a–c) and 2D U-87 (panels d–f) cells treated with NF-TMZ and NF-t, at 24 h (panels a–d), 48 h (panels b–e), and 72 h (panels c–f). 2D U-118 (panels g–i) and 2D U-87 (panels j–l) cells treated with TMZ at different concentrations, at 24 h (panels g–j), 48 h (panels h–k) and 72 h (panels i–l). Cell viability was evaluated by the PrestoBlue cell viability assay, and the results expressed as a percentage of untreated control cells. Values are the means ± SEM of triplicate analysis.

To establish the time and concentration cytotoxicity profile, 2D U-118 and 2D U-87 cells were treated with NF-t carrying TMZ at different concentrations (from 2.5 to 10 μM) for 24, 48, and 72 h and tested using the PrestoBlue cell viability assay. All treatments yielded statistically significant results with nanofibers carrying TMZ demonstrating a reduction in cell proliferation compared with the control. In both cell lines, the NF-t (100 μM) showed no toxic effects, indicating that the delivery platform developed represents a valuable tool for applications against various pathologies (Figure 6).

2D U-118 cells were significantly affected in viability by treatments with nanofibers conjugated to TMZ at 24, 48, and 72 h (Figure 6, panels a–c). When the concentration of TMZ was 10 μM, cell viability is about 78% of the control after 24 and 48 h treatments and 58% of the control after 72 h of treatment. In contrast, 2D U-87 cells were not affected at all at 24 h (Figure 6, panel d), while the effect was significant after 48 h treatment at the highest TMZ concentration (Figure 5, panel e) and after 72 h treatment at all tested TMZ concentrations (Figure 6, panel f).

We also analyzed the effect of free TMZ in our experimental setting at concentrations of 10, 100, and 250 μM. All TMZ treatments gave a statistically significant result, reducing cell proliferation compared to the control, although we observed low activity at 10 μM; furthermore, the addition of DMSO (0.133 M, used to dissolve TMZ) in the culture medium did not affect cell viability.

U-118 cell viability was affected by the treatments with free TMZ at 24, 48, and 72 h (Figure 6, panels g–i). When the concentration of TMZ was 10 μM, the cell viability ranged between 89 and 87% of the control in the three time treatments. U-87 cells were found to be much more resistant than U-118 to TMZ. With the 24- and 48-h treatments, TMZ acted on cell survival only at concentrations equal to or greater than 100 μM (Figure 6, panels j, k).

In 2D experiments, we observed a remarkable effect on U-118 cells with all nanofibers carrying TMZ at different concentrations, which increased proportionally with the concentration. In particular, treatment with NF-TMZ (carrying TMZ at a concentration of 10 μM) caused a decrease in cell viability at 72 h by more than 40%, whereas a similar decrease (under the same experimental conditions) is reported in the literature with a TMZ concentration of 100 μM.^41^ U-87 cells responded less to the treatment, but we still observed a reduction in cell viability of 30%. Indeed, the experiments performed at various concentrations of free TMZ showed that we obtained the same effect at a TMZ concentration between 100 and 250 μM. In contrast, in 3D experiments, both cell types showed an increase in the activity of free TMZ already at 10 μM. It is important to note that 2D and 3D systems are not easily comparable due to significant differences in culture conditions, physiological environments, and cell–cell and drug–cell interactions. In 3D cultures, the cells’ three-dimensional arrangement consists of an outer proliferating layer, a middle quiescent layer, and a central region, which produces different levels of exposure to drugs. These differences can lead to varying cell behavior upon exposure to the same drug.

**Figure 7:**
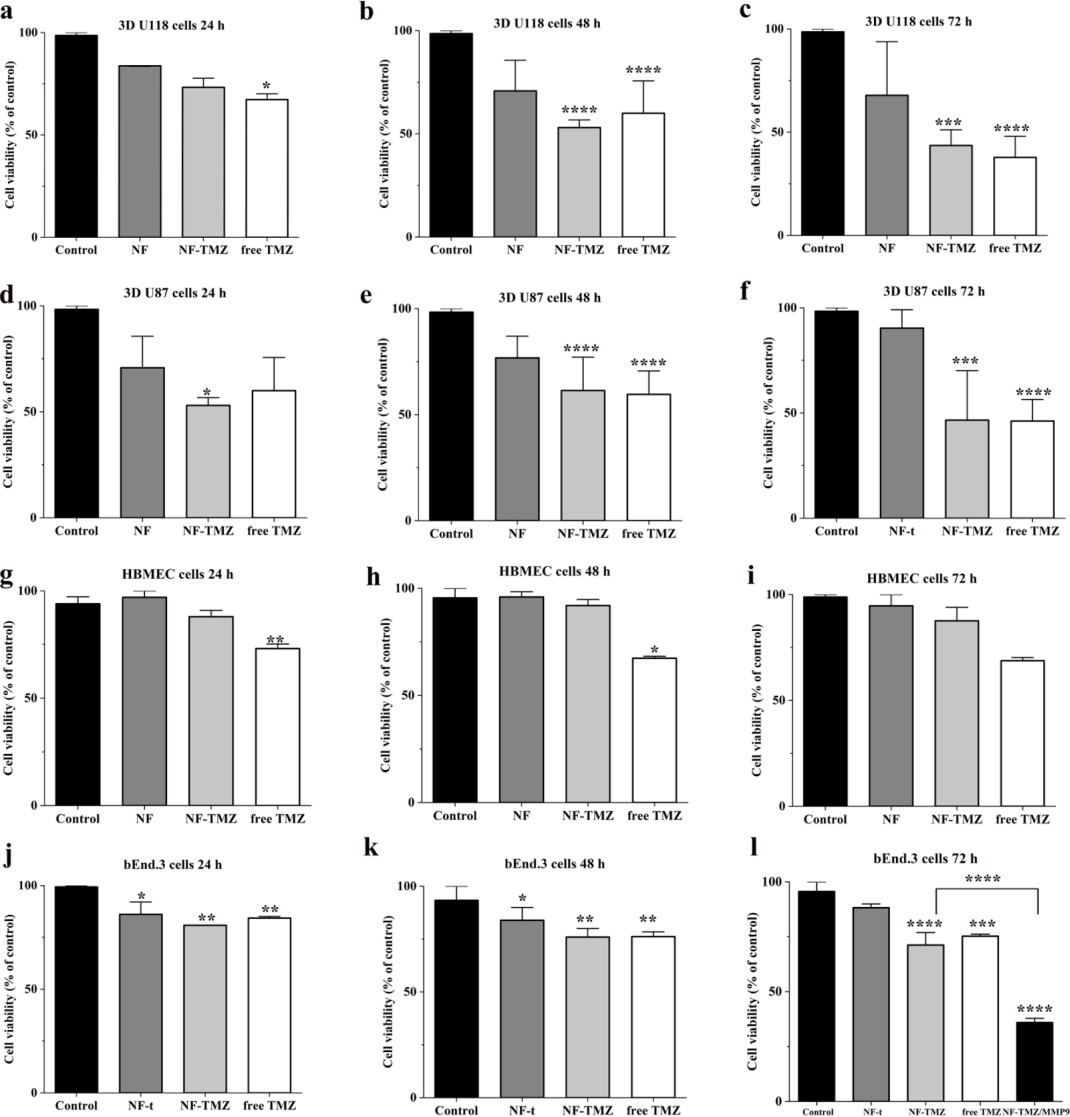
Panels a–f show 3D U-118 and 3D U-87 PrestoBlue cell viability assay. Panels g–i show the HBMEC PrestoBlue cell viability assay. Panels j–l show bEnd.3 cells PrestoBlue cell viability assay. 3D cells are treated with of NF-t; NF-TMZ (10 μM), and free TMZ (10 μM). Values are the means ± SEM of triplicate analysis.

After performing a 2D cell viability assay, we evaluated viability in 3D cells at 24, 48, and 72 h. We obtained no toxicity after treatment with the NF-t without the drug for up to 72 h in both 3D U-87 and 3D U-118 cells. 3D U-118 treated with NF carrying 10 μM TMZ (NF-TMZ) showed a decrease of ∼50% compared to the control at 48 h. This result is particularly interesting since it clearly demonstrates that the drug is active, although being covalently bound to the surface of the nanofiber, and the fiber does not interfere with its activity (Figure 7a–c). Also, 3D U-87, after 72 h of treatment, showed cell viability values for NF-t comparable to the control, indicating that the platform is not toxic to this cell line. 3D U-87 cells treated with NF carrying 10 μM TMZ (NF-TMZ) showed a decrease in viability of 65% compared to the control after 72 h. 3D U-87 cells treated with 10 μM TMZ showed a decrease in viability of 55% compared to the control at 72 h (Figure 7d–f).

To verify whether NF-t or NF-TMZ influenced healthy brain endothelial cells constitutively expressing EGFRs, we performed an HBMEC cell viability assay. HBMEC cells were treated every 24 h for 3 days with TMZ (10 μM) conjugated to the fiber and not conjugated to the fiber. The control was represented by cells cultured in the appropriate medium. Cell viability evaluated with the PrestoBlue assay shows no significant difference among the different experimental classes. This indicates that the NF-t, the NF-TMZ, and free TMZ do not act on healthy cells constitutively expressing EGFRs. Moreover, HBMEC are brain endothelial cells that obstruct TMZ passage; as a matter of fact TMZ does not affect HBMEC as confirmed by literature data^43,44^ (Figure 7, panels g–i).

NF-TMZ (10 μM) and TMZ (10 μM) effects were evaluated on another brain endothelial cell line: bEnd.3 (Figure 7, panels j and k). The results clearly showed a similar effect to that of HBMECs after 72 h of treatment. Next, we sought to evaluate whether the TMZ could be released intracellularly by bEnd.3 cells by performing an experiment in which NF-TMZ was pretreated or not with the MMP-9-activated enzyme and then verifying whether we observed a significant reduction in cell viability at 72 h (Figure 7, panel l). When fibers were pretreated with MMP-9 before incubations, the toxicity on bEnd.3 cells increased significantly. In fact, we observed a decrease in viability of 64% compared with the control, while NF-TMZ not treated with MMP-9 showed a decrease of 29%. Overall, these results support the evidence that this strategy is highly efficient for drug on-demand release and confirm the results described above obtained by UV–vis spectroscopy.

### 3.7 Analysis of Necrotic versus Apoptotic Cells

Annexin V-FITC and Propidium Iodide (PI) assays were performed in 3D U-87 and 3D U-118 cells to discriminate necrotic from apoptotic cells after 24 h treatment with NF-t, NF-TMZ (10 μM), and free TMZ (10 μM). Annexin V (labeled with FITC) determines the green color of apoptotic cells, while PI colors in red the necrotic cells.

**Figure 8:**
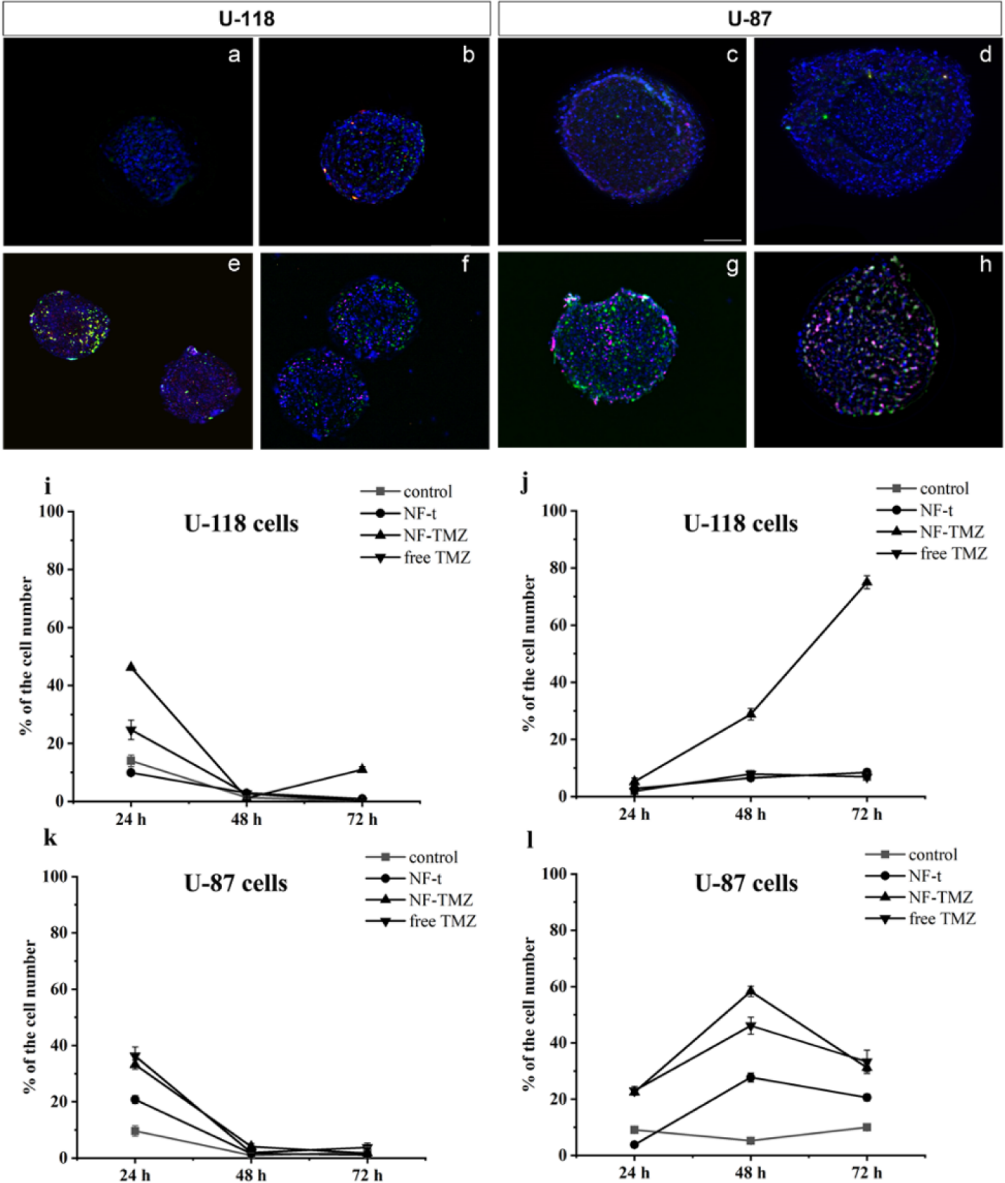
(a–h) Representative images of Annexin V and PI staining on U-118 (a, b, e, f) and U87 (c, d, g, h) spheroids before (a–c) and after treatment with NF-t (b–d), NF-TMZ (e–g), and free TMZ (f–h). All spheroids were labeled with DAPI to count total cell number. Scale bar 100 μm. (i–n) Annexin V (i, k) and PI (j,l) time course evaluation on control and treated spheroids. (i, j) U118 and (k,l) U-87spheroids treated with NF, NF loaded with 10 μM of TMZ, and 10 μM of TMZ. Data are expressed as percentage of the total cell number.

3D U-87 and 3D U-118 Annexin V/PI analyses are reported in Figure 8, (i–l). The evaluation of the apoptotic profile (24, 48, and 72 h) showed the same decreasing trend (Figure 8, panels i, k) for both cell lines. In contrast, necrotic cells showed a sharp difference between the cell lines. Necrotic cells decreased in number in U-87 spheroids at 72 h (Figure 8, panel l), while a significant increase in necrotic cells was observed in U-118 spheroids up to 72 h when treated with NF-TMZ (Figure 8, panel j).

In detail, 3D-U118 expressed low levels of apoptotic cells for the control and NF-t at 24 h; furthermore, we observed an initial burst of apoptosis when we treated cells with NF-TMZ (10 μM) and free TMZ (10 μM) (Figure 8, panel (i)). The same trend was observed for the 3D-U87 control and NF-t, while a four-time increasing level with NF-TMZ (10 μM) and free TMZ (10 μM) treatment was observed compared to the control. At 48 h, we observed a decrease in apoptotic cells for both 3D cell types (Figure 8, panel k).

As for necrotic cells, a different pattern was observed. In particular, for U-118 spheroids, their number always remained low except for spheroids treated with NF-TMZ, where we observed a significant increase throughout the entire experiment (Figure 8, panel j). For 3D U-87, we observed an increase in necrotic cells in the treated cells (NF-TMZ and free TMZ) at 48 h (Figure 8, panel l).

Indeed, we observed both increased apoptotic and necrotic cells in the sample treated with NF-TMZ and a huge increase in necrotic U-118 cells compared to free TMZ at the same concentration, indicating that the platform is highly effective.

**Figure 9:**
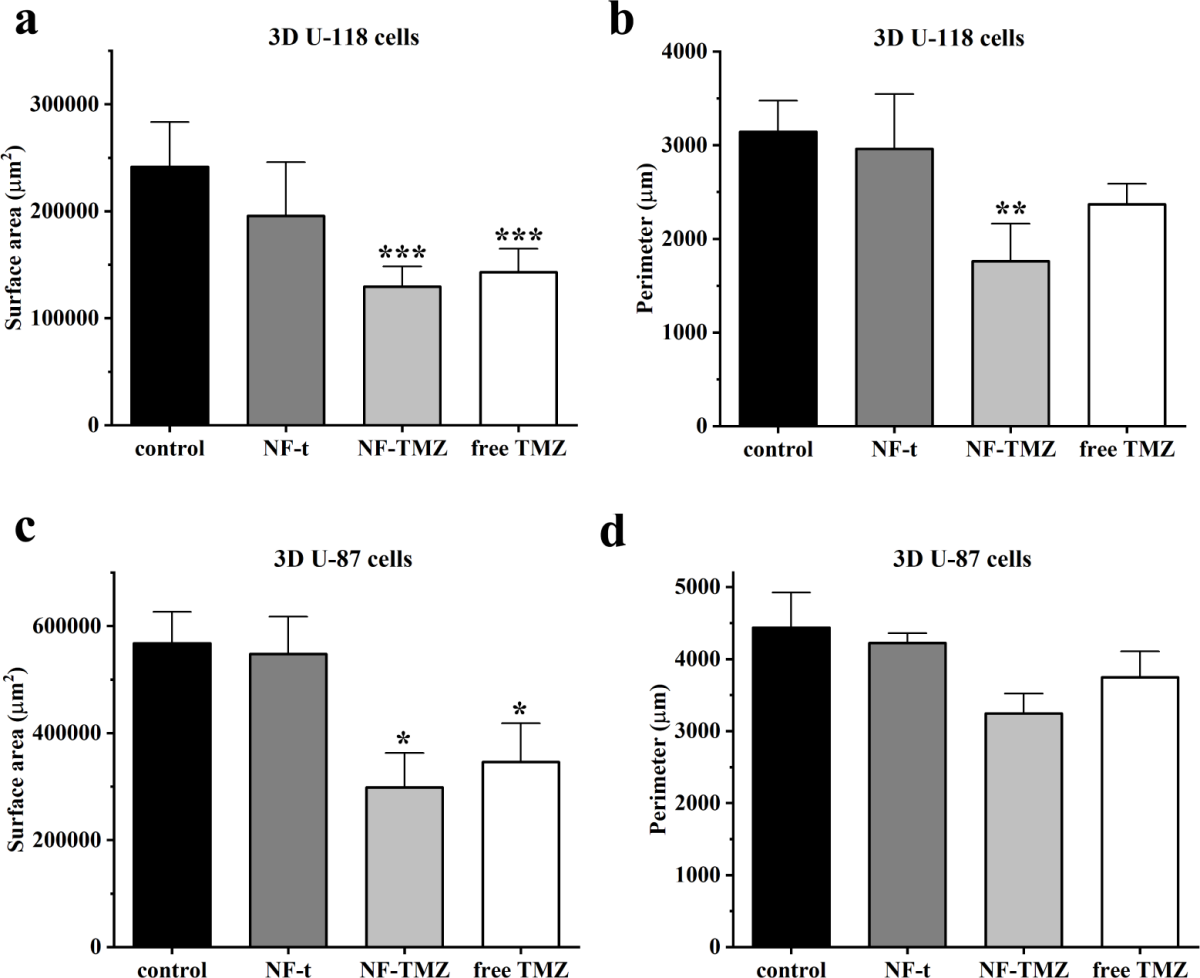
Surface area and perimeter of U118 MG (a, b) and U87 MG (c, d) spheroids after treatment. ***p < 0.001, **p < 0.01 compared to control.

To evaluate shape descriptors, 10 spheroids for each formulation were imaged and analyzed by Fiji software (Table 4). Surface area was significantly lower for both 3D U-118 and 3D U-87 after NF-TMZ and free TMZ treatment compared to the control and NF-t (Figure 9, panels a, c). The perimeter of spheroids was significantly lower for NF-TMZ treatment on U-118, exclusively (Figure 9, panels b, d). Feret’s diameter was significantly lower for NF-TMZ-treated U-118 cells (Table 4).

**Table 4:** Shape Descriptors of Spheroids U118MG and U87 MG

U118 MG	Circularity	Feret’s diameter	Min Feret	AR	Round
Controls	0.286	659.166	531.858	1.194	0.842
NF-t	0.384	571.450	486.575	1.139	0.879
NF+TMZ	0.626	461.009***	401.514***	1.113	0.903
Free TMZ	0.425	512.096	397.785	1.298	0.802

U87 MG	Circularity	Feret’s diameter	Min Feret	AR	Round
Controls	0.432	948.758	720.450	1.317	0.779
NF-t	0.267	996.822	784.555	1.225	0.825
NF+TMZ	0.352	879.891	669.086	1.299	0.794
Free TMZ	0.280	841.910	612.895	1.404	0.732

Table 4 was obtained using Fiji software, for U-118 and U-87 spheroids treated with NF-TMZ, free TMZ, and NF-t, compared with controls (no treatment), ***p < 0.001. Circularity was calculated as . Values of 1.0 indicate a perfect circle; while as the value approaches 0.0, it indicates an increasingly elongated shape. Feret’s diameter was the maximum caliper of the object, also known as the longest distance between any two points along the selection boundary. The minimum caliper diameter is indicated as Min Feret. The aspect ratio of the particle’s fitted ellipse was calculated as  and is indicated as AR. Roundness was calculated as 4  and is the inverse of AR. Shape descriptors describe the eventual changes in spheroid morphology following treatment.

### 3.8 The BBB Crossing of NF-TMZ and Its Cytotoxicity on 3D Dynamic In Vitro BBB Model

**Figure 10:**
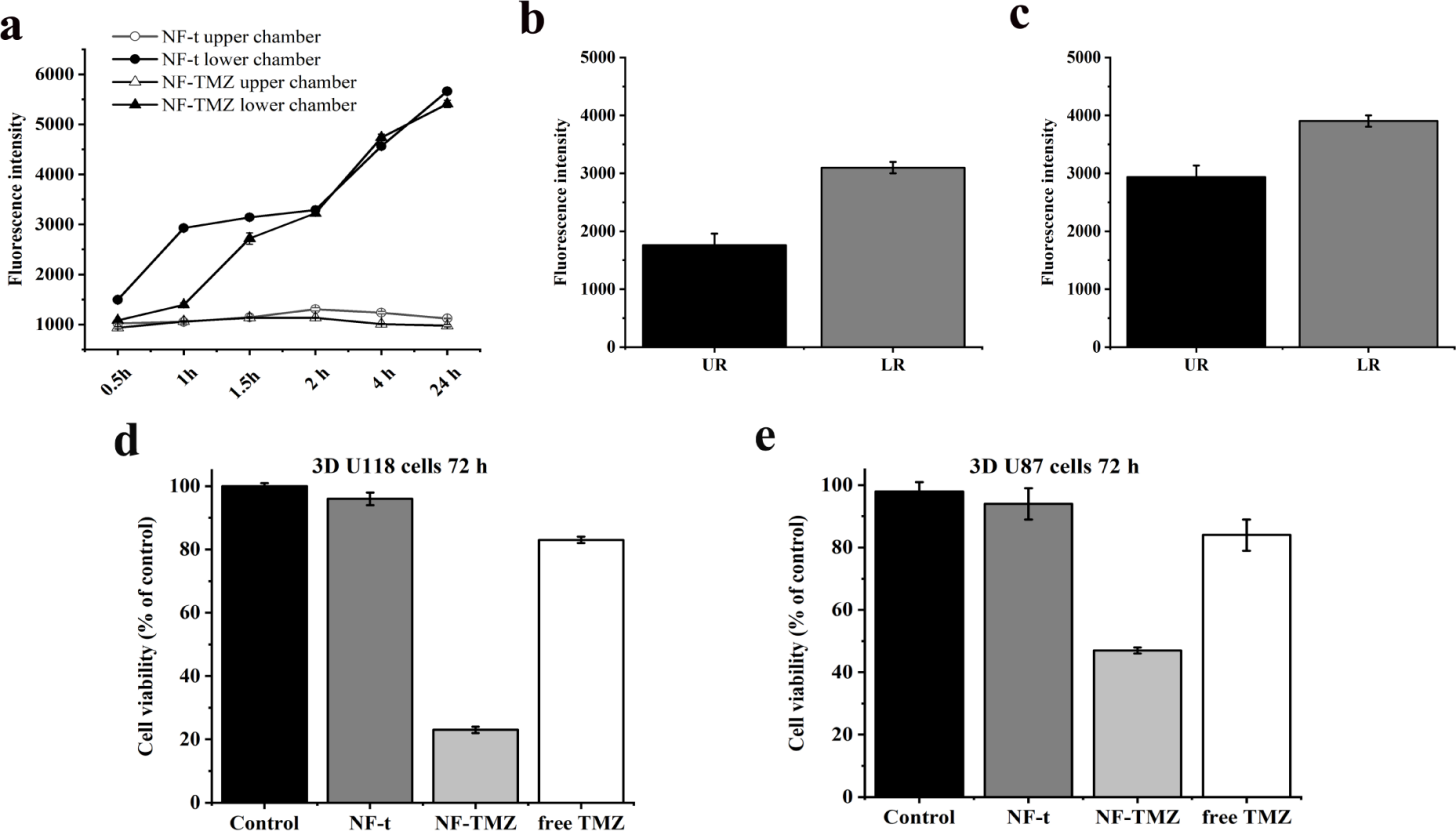
Panel a: Spectrofluorometric analysis of FITC-NF-t and FITC-NF-TMZ delivery across BBB dynamic *in vitro* model from 0.5 to 24 h. Data ± SEM. Panel b: After 24 h of FITC-NF-TMZ injection, the passage inside the upper reservoir and lower reservoir (LR) were then evaluated. Data ± SEM. Panel c: After 24 h of FITC-NF-t injection, the passage inside the UR and LR were then evaluated. Panels d and e: PrestoBlue cell viability assay in an *in vitro* BBB fluid-dynamic model on 3D U-118 (d) and 3D U-87 (e). 3D cells are treated with of NF-t, NF-TMZ (10 μM), and free TMZ (10 μM). Data ± SEM. ****p < 0.0001; ***p < 0.001.

We evaluated the cytotoxicity of NF-TMZ after the passage of FITC-NF-TMZ through the BBB model, as described above. First, we performed a spectrofluorimetric assay exploiting the LB2 bioreactor. Samples of medium were taken at regular intervals (0.5, 1, 1.5, 2, 4, and 24 h) in the outlets of both chambers (upper and lower). There was an increase in FITC-NF-TMZ fluorescence in the lower chamber (LC) compared to the upper camera (UC) starting from 1 h up to 24 h (Figure 10). The same trend was observed for FITC-NF-t (Figure 10). This is indicative that TMZ does not have an impact on the mechanism of internalization, and the trend of the increase is similar with and without TMZ.

Moreover, at the end of the experiments, we can clearly observe a more consistent increase in total fluorescence in the lower reservoir (LR) compared to the upper reservoir (UR) of LB2 after the passage of FITC-NF-TMZ (Figure 10, panel b). The same trend was observed for FITC-NF-t reservoirs (Figure 10, panel c). As in the previous experiment, this is indicative that TMZ does not lower the activity of gH bound to the fiber but maintains a consistently high trend from 1 to 24 h.

After evaluating the effective passage of NF-TMZ through the 3D dynamic *in vitro* BBB model, we assessed its effect on 3D U-87 and 3D U-118 cells located in the lower chamber. We developed different bioreactors containing in the upper chamber of HBMEC and hPC-PL cells and in the lower chamber and 3D U-118 cells or 3D U-87 cells. We injected NF-TMZ, NF-t, and free TMZ and after 72 h, we performed a PrestoBlue cell viability assay. For the 3D U-118 bioreactor, we observed about 80% lower viability for NF-TMZ treatment compared with the control. Interestingly, after the injection of free TMZ, cells showed less than a 20% decrease in viability. The same trend was observed for the 3D U-87 bioreactor, with 50% lower viability after NF-TMZ injection and 13% lower viability after the injection of free TMZ. Furthermore, no decrease was observed after the injection of NF-t. These data clearly support the view that the nanofiber helps the passage of TMZ into the lower chamber determining a lowering of cell viability (Figure 10, panels d,e).

## 4 Discussions

The goal of this work was to develop a nanoplatform based on self-assembling peptides whose surfaces were covalently functionalized to have a targeted release of TMZ. This was achieved through a targeting peptide that binds to EGFRvIII, which is overexpressed on tumor cells, combined with an on-demand drug release strategy for enhanced therapeutic precision. First, we characterized the nanofibers obtained for their stability, CAC, and morphology, and then we evaluated their effect in both 2D and 3D U-87 and U-118 cell lines derived from the human brain.

In 2D experiments, we observed a remarkable effect of NF-TMZ (TMZ 10 μM) on both U-118 and U-87 cells; a similar effect (under the same experimental conditions) was found with a free TMZ concentration of 100–250 μM. This is of interest since it suggests that we were able to achieve the same pharmacological effect with at least a 10-fold lower concentration compared with routine treatments. 3D experiments, carried out for up to 72 h of treatment on 3D U-118 and 3D U-87 cells, showed a significant decrease in cell viability, and similar effects were obtained when the spheroids were treated with free TMZ at a concentration of 10 μM. These data indicate that our nanoplatform is effective in inducing a pharmacological effect on GBM cells. Furthermore, we are confident that our nanoplatform possesses a good safety profile since the nanofiber alone (both NF and NF-t) exhibits values almost comparable to those of the control, thus not being toxic to the cells. Moreover, after treatments with Annexin V/PI (24 h), we observed both for 3D U-87 and 3D U-118 cells an increase in necrotic and apoptotic cells, especially in favor of the latter when the cells were treated with NF-TMZ (10 μM) and with free TMZ (10 μM). These data are in good agreement with the morphological analysis showing that treatments induce consistent changes in the histoarchitecture of the spheroids. As concerns U-118, spheroids were reduced in surface area, perimeter, and Feret’s diameter (used as an estimation of the spheroid size) when treated with NF-TMZ (10 μM). This suggests a loss of cells rather than the loss of a portion of the spheroid, which is also supported by the nonvariation of the AR aspect ratio (no statistically significant differences in elongation parameters of spheroids), coherently with the effectiveness of the nanoplatform. As concerns U-87 spheroids, the statistically significant decrease in surface area indicates an effect of the treatment, although there was a nonstatistically significant decrease in shape parameters. These data are also consistent with the vitality data, where the effects of the NF-TMZ (10 μM) or free TMZ (10 μM) treatments on the 3D U-87 have less impact on the spheroid’s viability. This could suggest that the different responses to treatments between cell lines could be due to phenotypical and/or genotypical specific features of GBM cell lines.^45^

The developed NF-TMZ has been demonstrated to permeate the BBB *in vitro* and to exhibit excellent penetration capabilities in a reliable *in vitro* dynamic model. We tested the passage of nanofiber alone (both NF and NF-t) and in combination with TMZ (10 μM) through this model. The time-dependent passage of nanofiber in the LB2 bioreactor, through the HBMEC/hPC–PL bilayer in the upper chamber and its release in the lower chamber where 3D U-87 or 3D U-118 were cultured, showed an increase in the lower chamber compared to the upper chamber for the FITC-NF and FITC-NF-t. Finally, there were no cytotoxic effects of nanofibers (NF-t) on spheroids in the lower chamber, while we observed a significant decrease in viability when using NF-TMZ. Furthermore, our BBB/Glioblastoma *in vitro* dynamic model demonstrated that the nanofiber functionalized with gH625 (NF) is an efficient delivery tool, allowing the crossing of the BBB without any toxicity, thus suggesting a good safety profile for this delivery system. The NF-TMZ’s enhanced penetration compared to free TMZ was further supported by its increased cytotoxic efficacy against 3D U-87 and 3D U-118 cells accessible after BBB crossing.

Undoubtedly, our nanoplatform system possesses superior performance for delivery through the BBB, thanks to the presence of gH625 and the targeting peptide P2-t. Furthermore, the TMZ delivered exhibits detrimental effects on spheroids that are significantly greater than those observed with free TMZ.

Overall, this study shows that the developed platform would be ideal for enhancing brain drug delivery since its versatility makes it easy to tailor and adapt for the treatment of other complex neurological pathologies, including epilepsy, Alzheimer’s disease, and Parkinson’s disease. The approach consists of modifying the NF’s surface with targeting moiety, including the use of mitochondrial or nuclear targeting sequences or ligands directed at specific receptors. The drug can also be changed and selected *ad hoc* for specific targets and pathologies, for example, the NF can be tailored for the delivery of biological drugs such as RNA/DNA to overcome their limitations in the clinical field.

## 5 Conclusions

In this work, a rational design was performed for the construction and development of peptide-based nanofibers to have a selective delivery of TMZ with an on-demand strategy. Nanofibers decorated with several biological moieties, including the targeting peptide (P2-t), the cell-penetrating peptide (P3), and the peptide-bound drug (P2-d), were characterized both biophysically and biologically. Nanofibers showed good stability under different conditions, including the dilution effect, ionic strength, and pH environments, preserving the β-type conformation.

First, we demonstrated the ability of our nanofiber to cross the BBB performing *in vitro* studies in a 3D spheroidal biodynamic BBB model. In biological 2D and 3D experiments, we observed a significant effect on cell viability after treating both U-118 and U-87 cells with NF-TMZ (TMZ 10 μM), while the same effect was detected at higher concentrations (100–250 μM) of free TMZ. Moreover, the Annexin V/PI assay showed an increase in necrotic and apoptotic cells, and the morphological analysis proved that both U-118 and U-87 spheroids were smaller in surface area, perimeter, and Feret’s diameter after treatment with NF-TMZ.

Our findings open new opportunities for further studies aimed at evaluating the stability of the self-assembling peptide nanoplatform in blood, its effects on blood parameters and components, and its potential to penetrate the BBB and release its cargo *in vivo*. Furthermore, long-term studies will be necessary to assess the sustained efficacy and safety of the nanoplatform *in vivo*. Data from animal models will be crucial to strengthening the applicability and translational relevance of the developed platform.

## Supporting Information Available

HPLC chromatograms and ESI-MS spectra of peptides P1, P2, P3, P2-t, and P2-t (Figures S1–S12). EPR experiments of nanofibers composed of P1, P2, and P3 (Figure S13). Nanofiber’s uptake on GBM cells (Figure S14); TEM images of NF-TMZ (Figure S15). (PDF)

## Abbreviations

BBB: blood-brain barrier
bEnd.3: brain endothelial cells
CAC: critical aggregation concentration
CD: circular dichroism
EPR: electron paramagnetic resonance
GBM: glioblastoma
HBMEC: human brain microvascular endothelial cells
hPC-PL: human pericytes
NR: Nile Red
PAs: peptide amphiphiles
ThT: thioflavin T
TMZ: temozolomide
U-87: glioblastoma/astrocytoma cells
U-118: glioma cell lines
