# Supplementary material for: Engineering Multifunctional Peptide-Decorated Nanofibers for Targeted Delivery of Temozolomide across the Blood–Brain Barrier
Source: Mol Pharm. 2025 Mar 17;22(4):1920–38. doi: 10.1021/acs.molpharmaceut.4c01125 (PMC11979881; doi:10.1021/acs.molpharmaceut.4c01125)
Supplement: Supplementary file 1 — mp4c01125_si_001.pdf [file mp4c01125_si_001.pdf]

Engineering multifunctional peptide-decorated nanofibers for targeted delivery of temozolomide across the blood-brain barrier

Rosa Bellavita [a§], Teresa Barra [b§], Simone Braccia [a], Marina Prisco [b], Salvatore Valiante [b], Assunta Lombardi [b], Linda Leone [c], Jessica Pisano [b], Rodolfo Esposito [c], Flavia Nastri [c], Gerardino D'Errico [c,d], Annarita Falanga [e], Stefania Galdiero [a*]

[a] *Department of Pharmacy, School of Medicine, University of Naples Federico II, 80131 Napoli, Italy.*

[b] *Department of Biology, University of Napoli Federico II, Via Cintia, 80126 Naples, Italy.*

[c] *Department of Chemical Sciences, University of Napoli Federico II, and 4CSGI (Unit of Naples), Via Cintia, 80126 Naples, Italy.*

[d] *CSGI (Unit of Naples), Via Cintia, 80126 Naples, Italy.*

[e] *Department of Agricultural Science, University of Naples Federico II, Via Università 100, Portici, 80055 Portici, Italy*

*Email: sgaldier@unina.it

[§]*R.B. and T.B. contributed equally to this work*

Table of content

1. HPLC chromatograms and HR-MS spectra of peptides P1, P2, P3, P2-t, P2-d, P2-f (Figures S1–S12) 2-7

3. EPR experiments of NF composed by P1, P2, and P3 (Figure S13) 8

4. Cell fiber (1% and 6% of P2-t) uptake evaluation (Figure S14) 8-9

5. TEM images of NF-TMZ (Figure S15) 10

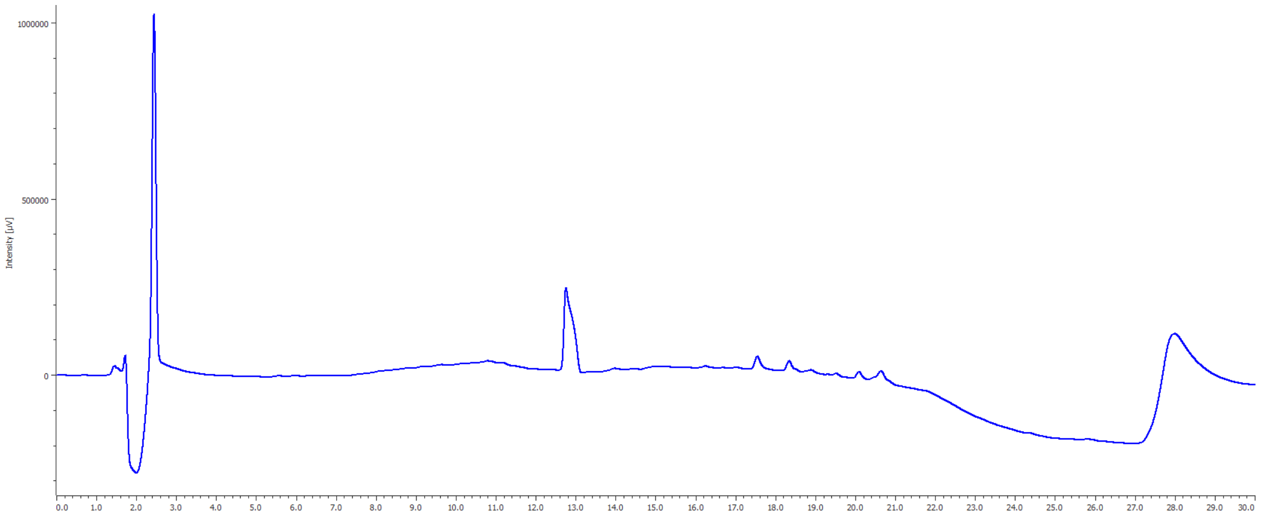

Figure S1. Chromatogram of **peptide P1** obtained by an analytical HPLC (Jasco LC-NetII/ADC) equipped with a Phenomenex Jupiter 4u Proteo column, 90 Å, 150 mm × 4.6 mm [linear gradient 10-90% MeCN (0.1% TFA) in H_2O (0.1% TFA) over 20 min, flow rate of 1 mL/min, and monitored by UV detection at 220 nm.

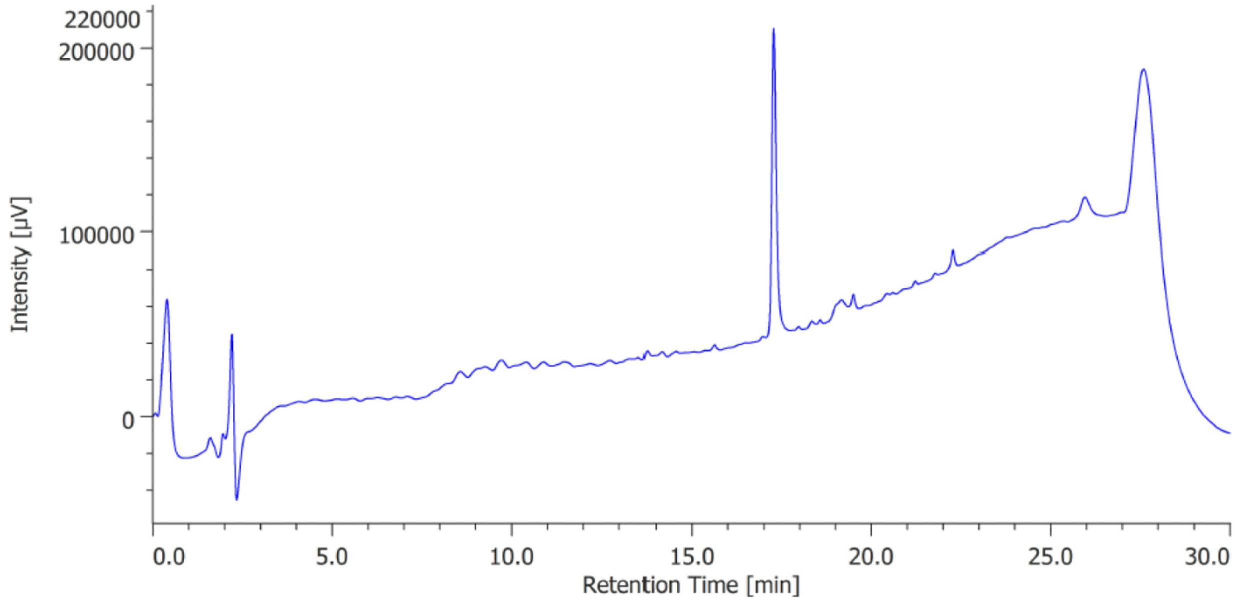

Figure S2. Chromatogram of **peptide P2** obtained by an analytical HPLC (Jasco LC-NetII/ADC) equipped with a Phenomenex Jupiter 4u (C12) Proteo column, 90 Å, 150 mm × 4.6 mm [linear gradient 10-90% MeCN (0.1% TFA) in H_2O (0.1% TFA) over 20 min, flow rate of 1 mL/min, and monitored by UV detection at 220 nm.

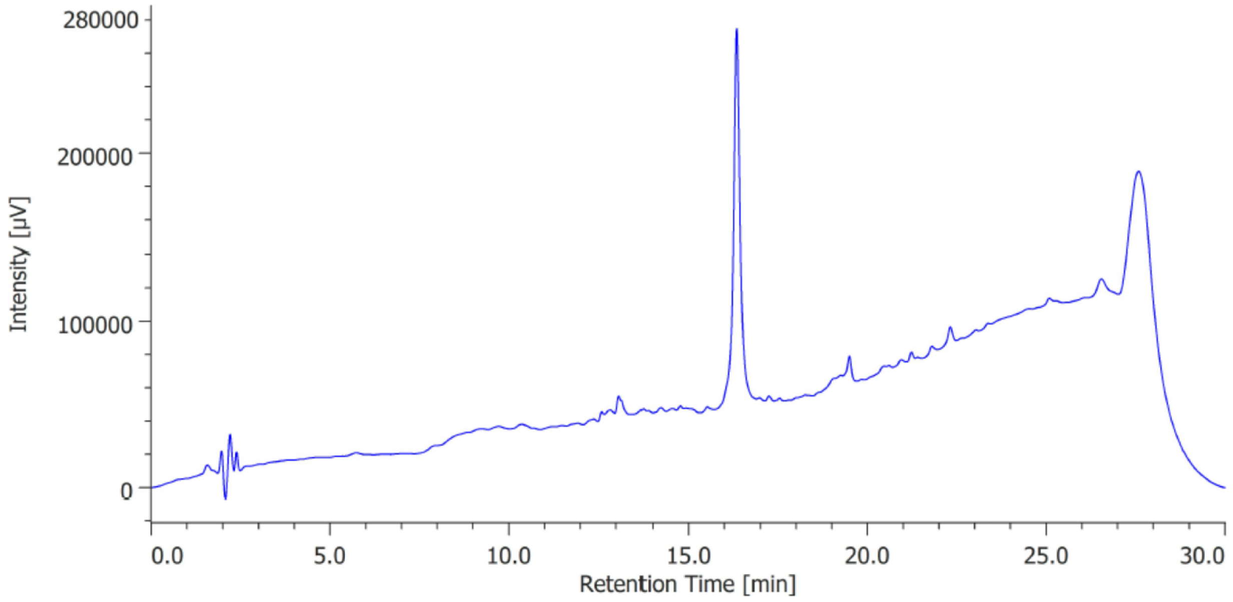

Figure S3. Chromatogram of **peptide P3** obtained by an analytical HPLC (Jasco LC-NetII/ADC) equipped with a Phenomenex Jupiter 4u Proteo column, 90 Å, 150 mm × 4.6 mm [linear gradient 10-90% MeCN (0.1% TFA) in H_2O (0.1% TFA) over 20 min, flow rate of 1 mL/min, and monitored by UV detection at 220 nm.

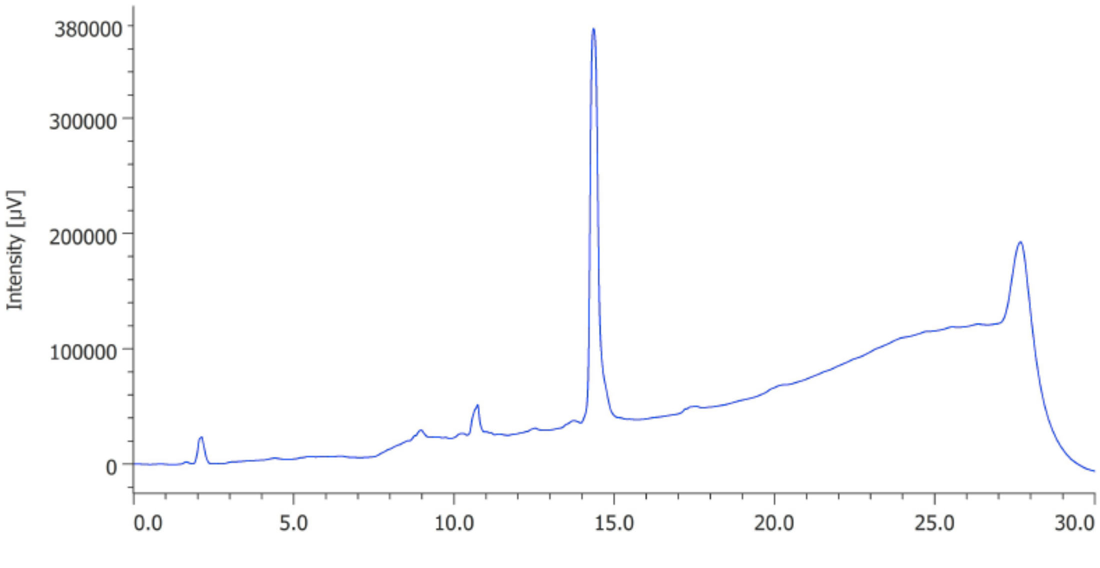

Figure S4. Chromatogram of **peptide P2-t** obtained by an analytical HPLC (Jasco LC-NetII/ADC) equipped with a Phenomenex Jupiter 4u Proteo column, 90 Å, 150 mm × 4.6 mm [linear gradient 10-

90% MeCN (0.1% TFA) in H_2O (0.1% TFA) over 20 min, flow rate of 1 mL/min, and monitored by UV detection at 220 nm.

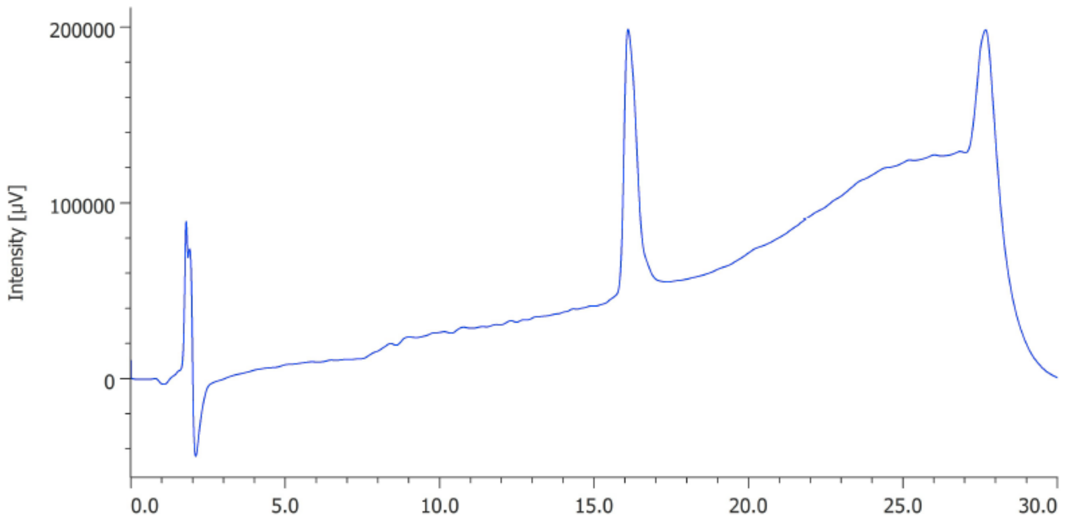

Figure S5. Chromatogram of **peptide P2-d** obtained by an analytical HPLC (Jasco LC-NetII/ADC) equipped with a Phenomenex Jupiter 4u Proteo column, 90 Å, 150 mm × 4.6 mm [linear gradient 10-90% MeCN (0.1% TFA) in H_2O (0.1% TFA) over 20 min, flow rate of 1 mL/min, and monitored by UV detection at 220 nm.

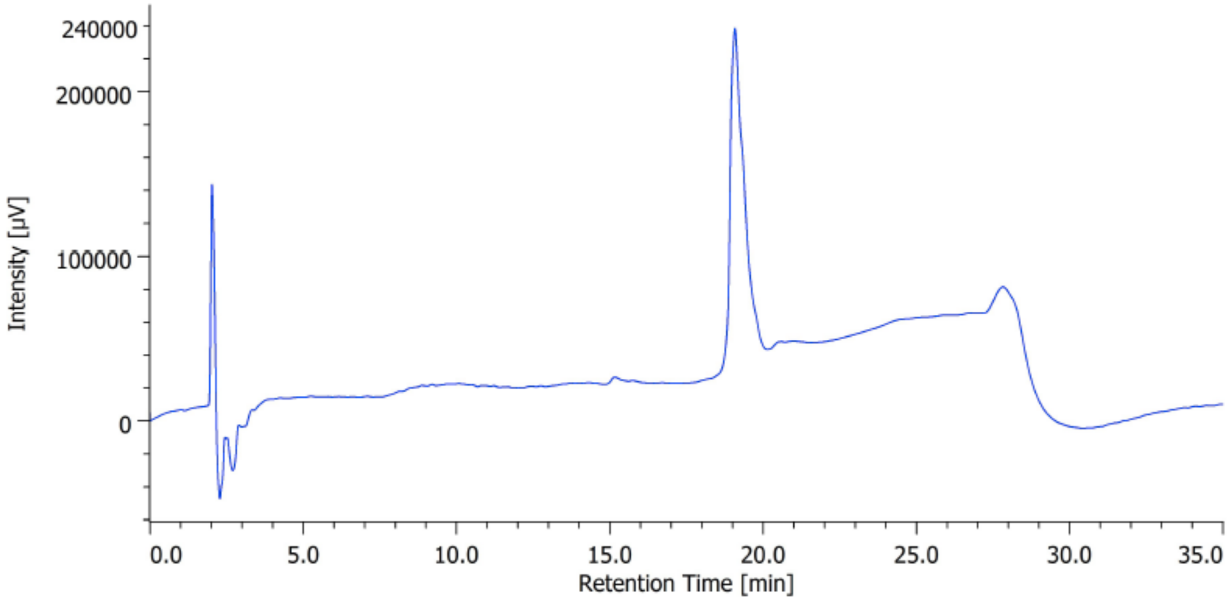

Figure S6. Chromatogram of **peptide P2-f** obtained by an analytical HPLC (Jasco LC-NetII/ADC) equipped with a Phenomenex Jupiter 4u Proteo column, 90 Å, 150 mm × 4.6 mm [linear gradient 10-

90% MeCN (0.1% TFA) in H_2O (0.1% TFA) over 20 min, flow rate of 1 mL/min, and monitored by UV detection at 220 nm.

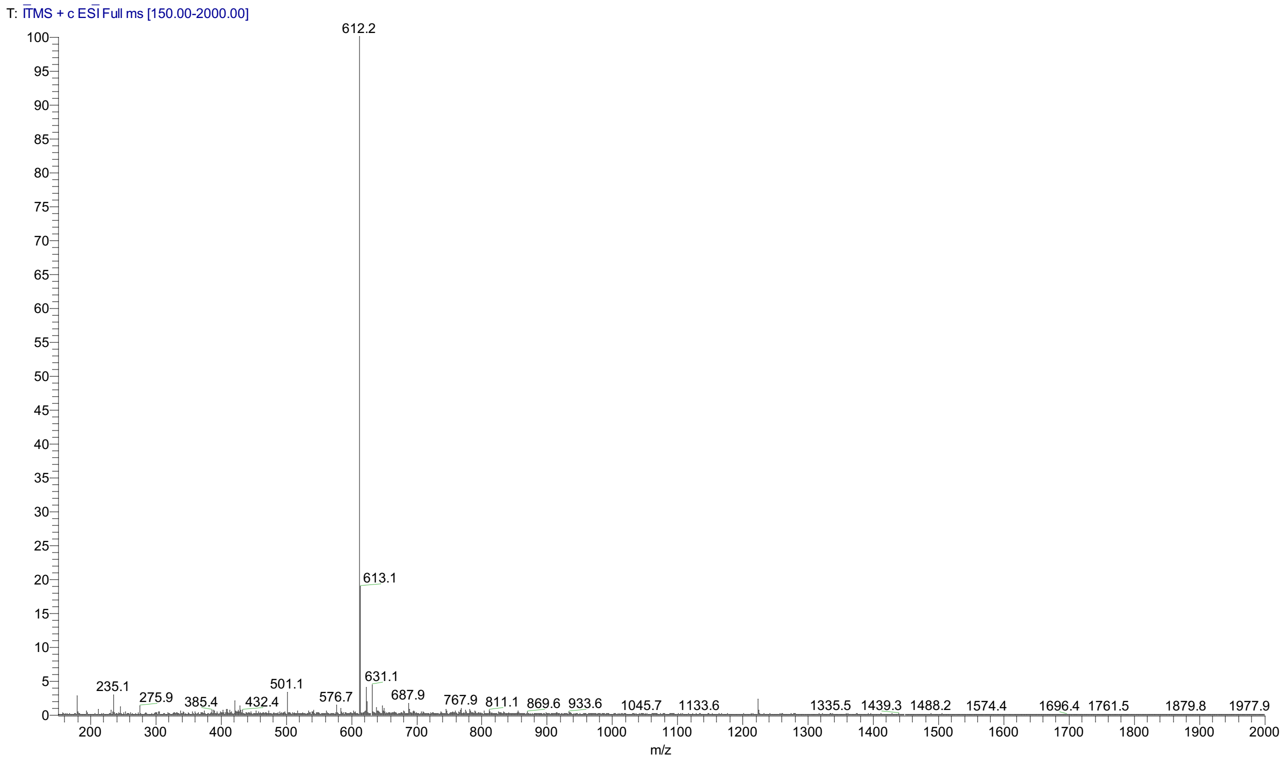

Figure S7. ESI-MS of peptide **P1**. Calculated mass: $[M\text{-}2H]^+/2$= 612.3. Found mass: $[M\text{-}2H]^+/2$= 612.1.

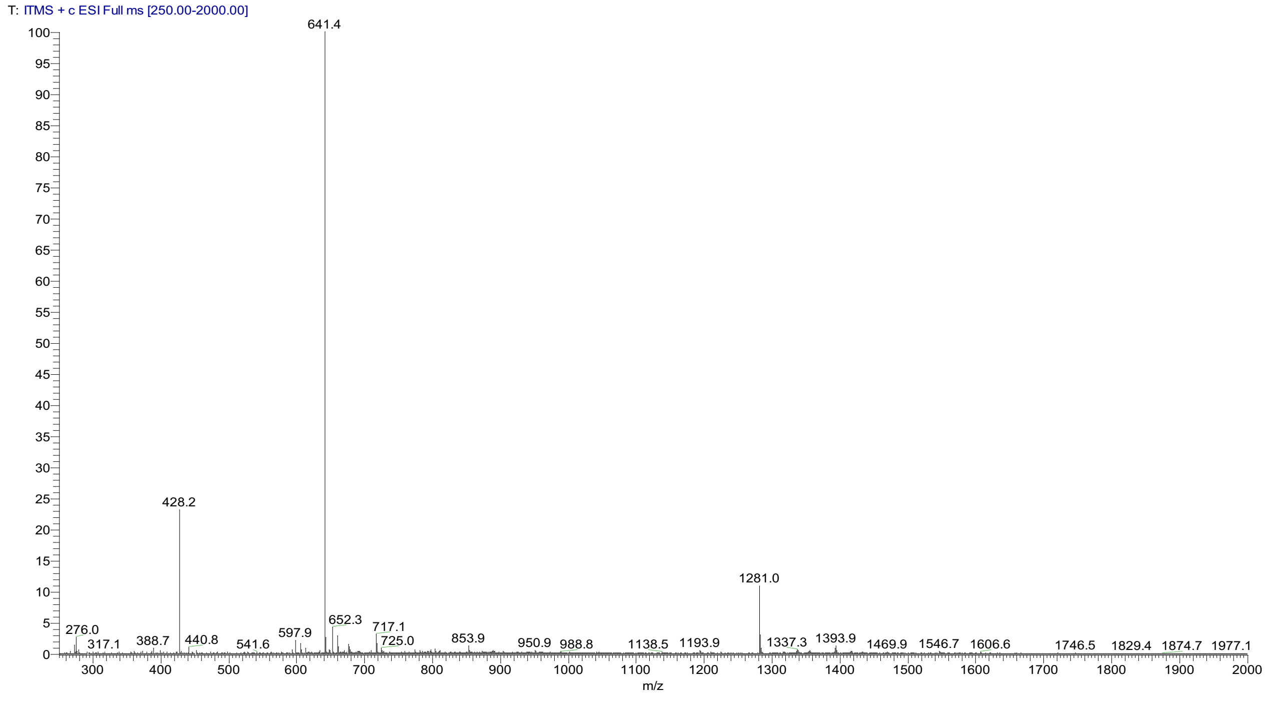

Figure S8. ESI-MS of peptide **P2**. Calculated mass: $[M+2H]^+/2$= 641.3. Found mass: $[M+2H]^+/2$= 641.4.

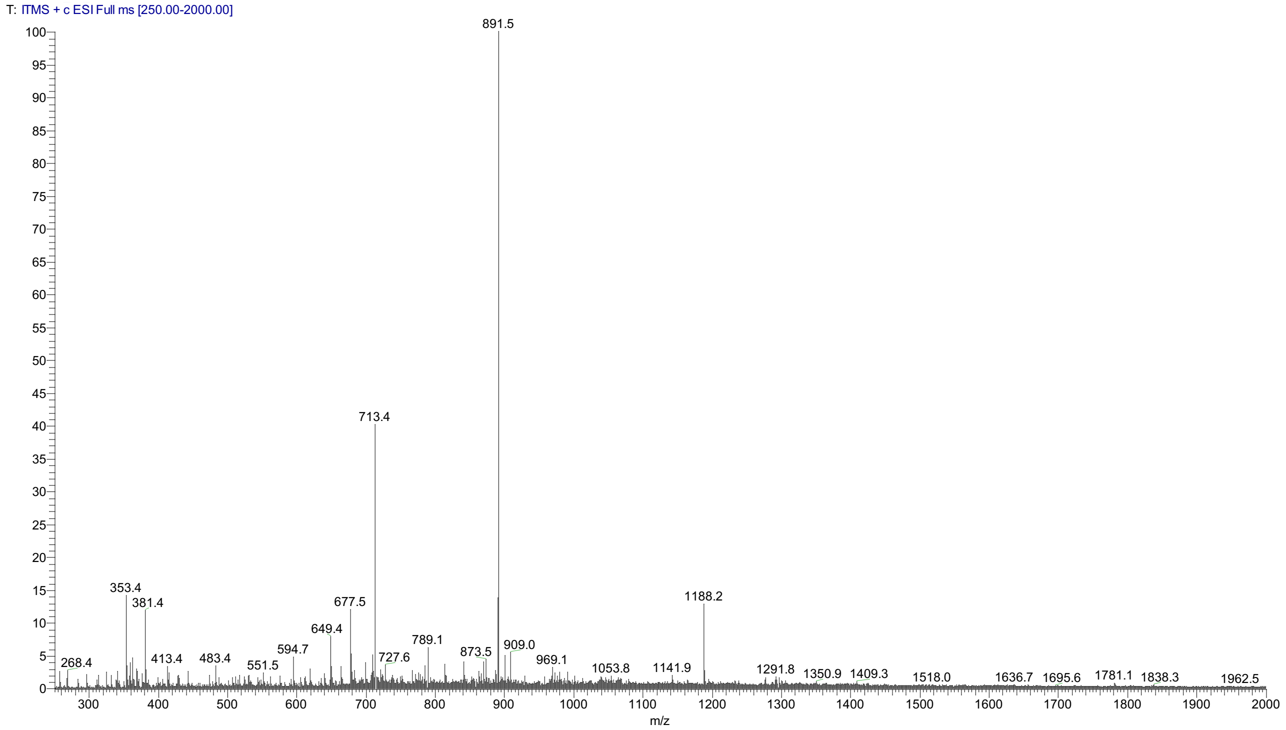

Figure S9. ESI-MS of peptide **P3**. Calculated mass: [M+3H]$^{+}$/3= 1188.1; [M+4H]$^{4+}$/4= 891.3. Found mass: [M+3H]$^{3+}$/3=1188.2; [M+4H]$^{4+}$/4=891.5.

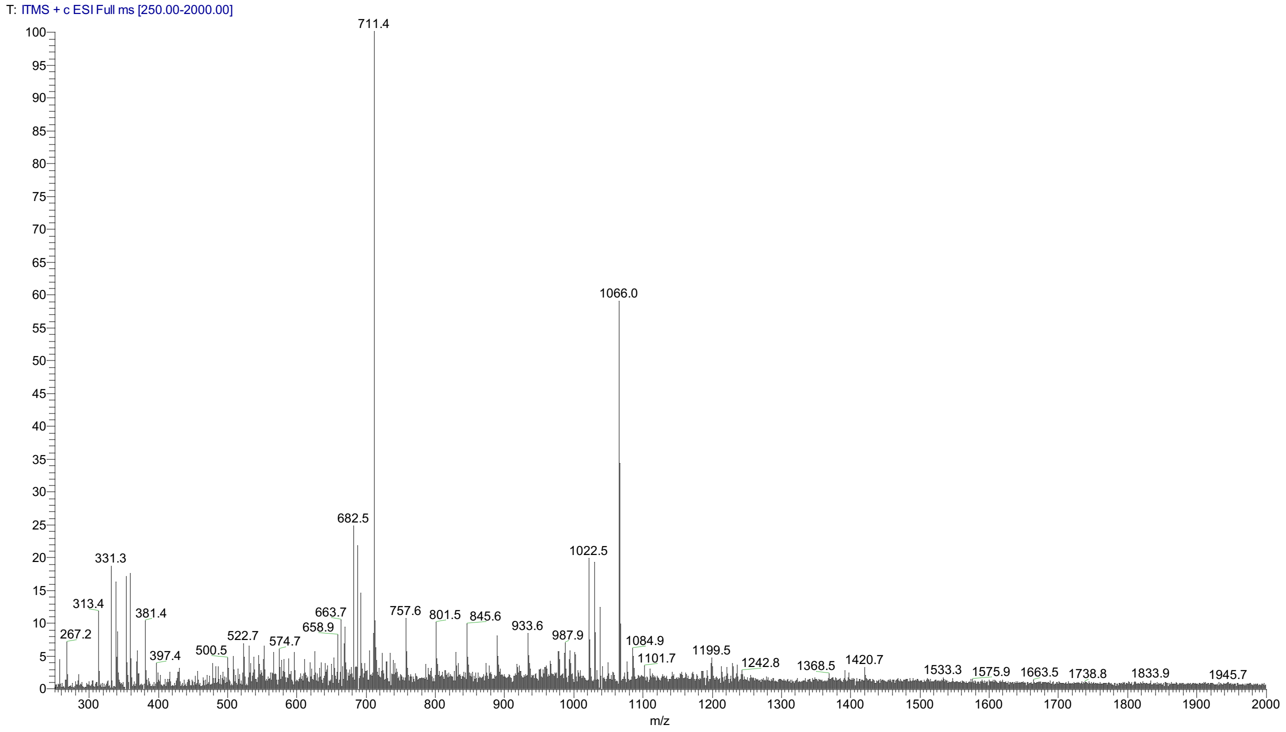

Figure S10. ESI-MS of peptide **P2-t**. Calculated mass: [M+2H]$^{+}$/2= 1066.3; [M+3H]$^{+}$/3= 711.2. Found mass: [M+2H]$^{+}$/2= 1066.1; [M+3H]$^{+}$/3= 711.4.

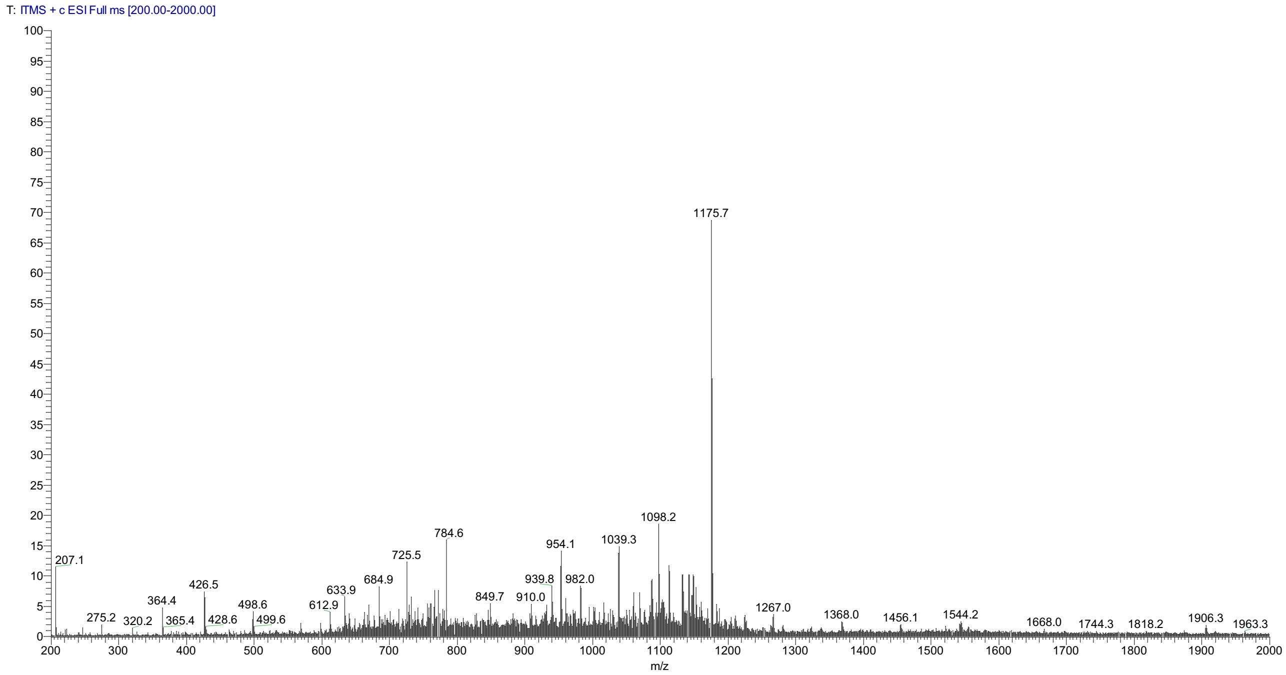

Figure S11. ESI-MS of peptide **P2-d**. Calculated mass: $[M+2H]^+/2$= 1175.5; $[M+3H]^+/3$= 783.9. Found mass: $[M+2H]^+/2$= 1175.7; $[M+3H]^+/3$= 784.6.

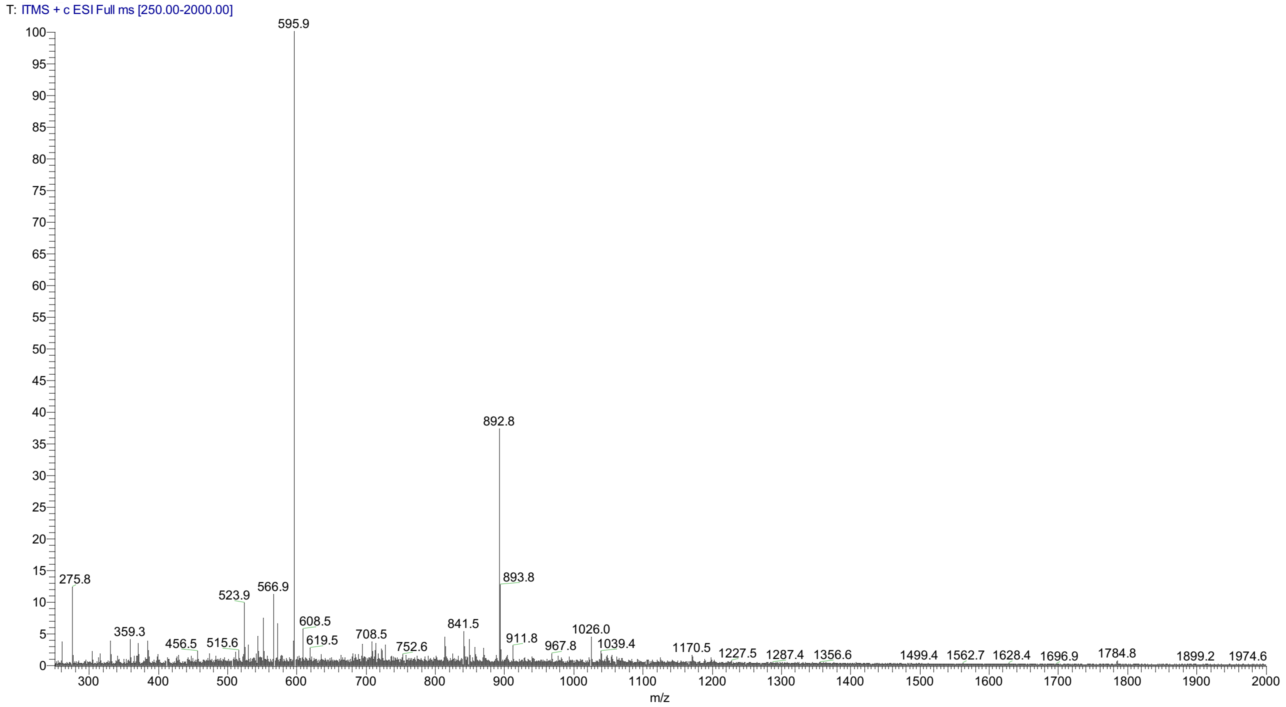

Figure S12. ESI-MS of peptide **P2-f**. Calculated mass: $[M+2H]^+/2$= 892.5; $[M+3H]^+/3$= 595.3. Found mass: $[M+2H]^+/2$= 892.8; $[M+3H]^+/3$= 596.9.

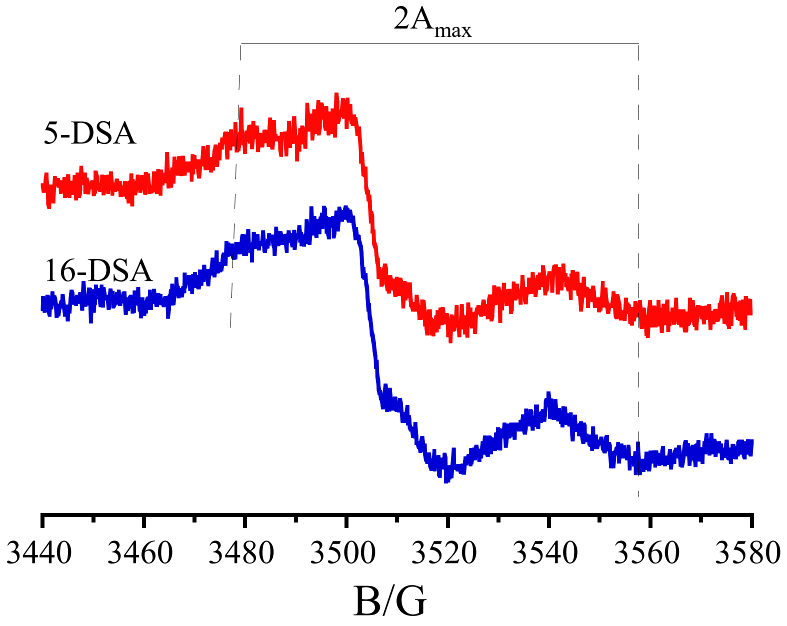

Figure S13. $2A_{max}$ values recorded for NF composed of P1+P2+P3 (0.5:0.45:0.05, molar ratio).

Cell fiber uptake evaluation. The cell uptake and cell morphology after treatments, were evaluated through fluorescence microscopy. We first determined the appropriate percentage of targeting peptide on the surface of the NF to obtain the acquisition of images of sufficient quality. We compared 1, 3 and 6% of targeting peptide on the surface of the NF and we decided to use 3% for GBM cell uptake experiments (Figure S14, panel a and b). The 1% sample is not easily observable and does not allow the acquisition of images of sufficient quality (Figure S14, panel b). The 3% sample is well observable and determines, under these conditions, a good quality of images, to evaluate the amount of fiber although treating GBM cells at 6% gives higher intensity results. We decided to use 3% percentage for further experiments to avoid possible fluorophore overloading of the fiber.

The standardized fluorescence measurement was obtained by spotting a 1/25 dilution of FITC 9μM and acquiring under the same conditions as the images of the treated cells.

a)

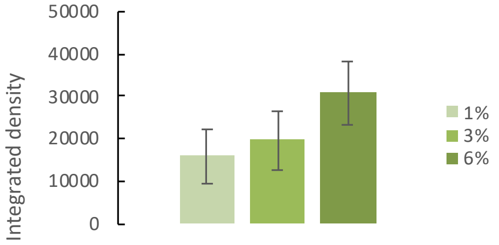

b)

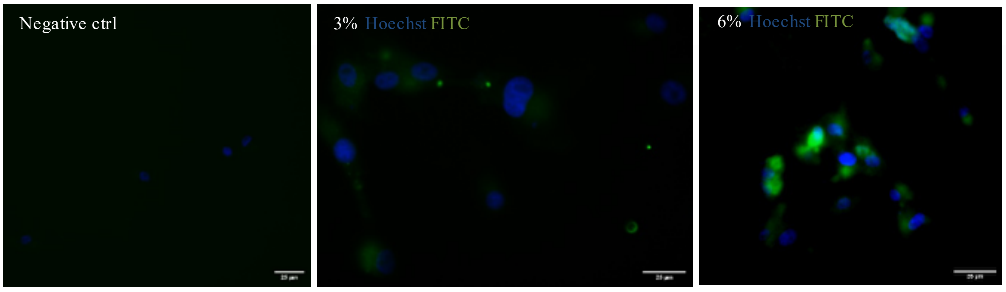

Figure S14. Panels a and b show that fluorescence in U118 increases proportionally with increasing percentage of the peptide P2-t.

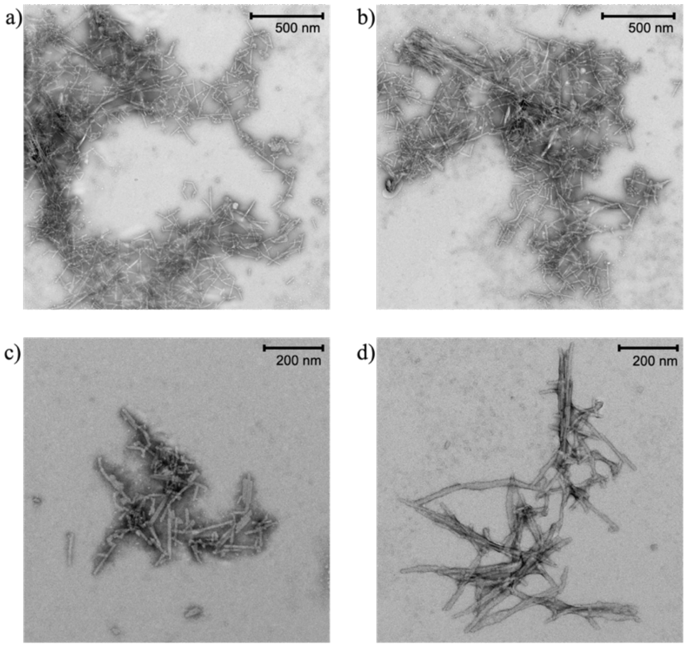

Figure S15. TEM image of NF-TMZ with different magnification scales. In panel a) and b), the scale bar represents 500 nm length, while in panel c) and d), the scale bar represents 200 nm.
